# Protein fibril length in cerebrospinal fluid is increased in Alzheimer’s disease

**DOI:** 10.1038/s42003-023-04606-7

**Published:** 2023-03-08

**Authors:** Peter Niraj Nirmalraj, Thomas Schneider, Lars Lüder, Ansgar Felbecker

**Affiliations:** 1 Transport at Nanoscale Interfaces Laboratory, Swiss Federal Laboratories for Materials Science and Technology, Dübendorf, CH-8600 Switzerland; 2 Department of Neurology, Cantonal Hospital St. Gallen, St. Gallen, CH-9007 Switzerland

**Keywords:** Dementia, Nanoscale biophysics

## Abstract

Alzheimer’s disease (AD) associated proteins exist in cerebrospinal fluid (CSF). This paper evidences that protein aggregate morphology distinctly differs in CSF of patients with AD dementia (ADD), mild cognitive impairment due to AD (MCI AD), with subjective cognitive decline without amyloid pathology (SCD) and with non-AD MCI using liquid-based atomic force microscopy (AFM). Spherical-shaped particles and nodular-shaped protofibrils were present in the CSF of SCD patients, whereas CSF of ADD patients abundantly contained elongated mature fibrils. Quantitative analysis of AFM topographs confirms fibril length is higher in CSF of ADD than in MCI AD and lowest in SCD and non-AD dementia patients. CSF fibril length is inversely correlated with CSF amyloid beta (Aβ) 42/40 ratio and CSF p-tau protein levels (obtained from biochemical assays) to predict amyloid and tau pathology with an accuracy of 94% and 82%, respectively, thus identifying ultralong protein fibrils in CSF as a possible signature of AD pathology.

## Abstract (web summary)

Liquid-based AFM on protein aggregates at single-fibril resolution from the cerebrospinal fluid of patients at various stages of AD reveals that mature fibrils from patients at advanced stage AD have longer length, distinct morphology and spatial organization.

## Introduction

CSF analytics is central in the diagnosis of neurodegenerative disorders including Parkinson’s disease (PD)^1^ and AD^2–9^. Chiefly, CSF reflects neuropathological changes upon interfacing with the extracellular brain regions, thus serving as a rich source for screening PD and AD biomarkers. Still, it has only been in the recent past that the quantification of Aβ42 and 40 and their ratio, total-tau (t-tau) and phosphorylated-tau (p-tau) protein levels in CSF using enzyme-linked immunosorbent assay (ELISA) have become a cornerstone of the routine diagnostics of patients with cognitive complaints as they allow for an early and reliable diagnosis of AD pathology^2,4,5,10,11^. In particular, the Aβ42 to Aβ40 ratio is decreased early in the progression of AD and is inversely correlated with plaque formation found on Amyloid-PET^3,12^, while phosphorylated tau protein levels are increased in AD patients and reflect disease severity^13,14^. The advent of digital immunoassays further enabled the realisation of an accurate assessment of pathologic amyloid and p-tau isoforms also in blood plasma^15–17^. Especially, Aβ42, P-tau181, P-tau217, and P-tau231 levels in plasma were shown to be highly correlated with CSF- and PET AD biomarkers^16–19^. Alternative techniques such as immuno-infrared sensors were demonstrated to reliably detect the formation of β-sheet structures indicative of pathologic Aβ-oligomerization^20^. Analysis of the changes associated with the secondary Aβ structure allowed the detection of AD pathology with a high accuracy^21^ even in preclinical and prodromal AD patients already up to 17 years before clinical conversion to AD^22,23^. Regarding the tertiary structure of insoluble Aβ aggregates, in the past, fluorescence correlation spectroscopy was demonstrated to identify amyloid-beta aggregates in CSF^24^ but it has been shown that fluorophore labels can modify oligomer size distribution^25^. Cryo-electron microscopy is another powerful tool employed to capture the polymorphism in brain-derived amyloid fibrils^26,27^ but the sample preparation steps required for protein fibril analysis at cryogenic conditions could affect the fibril structure^28,29^.

Although crucial qualitative and quantitative information on protein aggregates can be obtained from the above-discussed methods, it is becoming increasingly important to characterise single protein aggregates in CSF in a label-free manner under physiological conditions.

In this context, AFM, infrared spectroscopy and Raman spectroscopy are promising preclinical techniques to examine in a label-free manner the nanoscale morphology^30–32^ and chemical signature^33,34^ of oligomers and fibrillar aggregates of proteins involved in the pathogenesis of AD in CSF^30,31^ and in blood^35^. Clearly, AFM operated at ambient conditions cannot provide atomic-scale details on protein aggregates on par with cryo-electron microscopy (cryo-EM) but it offers a facile route to profile the full spectrum of pathological protein aggregates with single-particle specificty^36–39^ without the need for complex sample preparation. Knowledge on the differences in protein aggregate types in body fluids from small oligomers, and protofibrils to mature fibrils could augment current AD diagnostics and treatment decisions. For example, antibodies against Aβ that have been developed in the past years for the treatment of AD seem to target different protein aggregates of Aβ. In particular, the anti-amyloid antibody lecanemab, which mainly binds to soluble protofibrils (short fibrillar aggregates that appear with a distinct nodular morphology^35,36,40^) showed positive results on clinical endpoints in a large phase-III trial^41^. In contrast, previous phase III studies on anti-amyloid antibodies which mainly bind to mature amyloid fibrils (elongated with smooth surface topology) such as gantenerumab, crenezumab and aducanumab showed no (gantenerumab, crenezumab) or uncertain (aducanumab) effects on clinical endpoints^42,43^. Thus, the question of which kind of protein aggregates are most prevalent in body fluids such as the CSF of an individual patient could be of major importance for the selection of patients for clinical trials or treatment decisions.

**Fig. 1:**
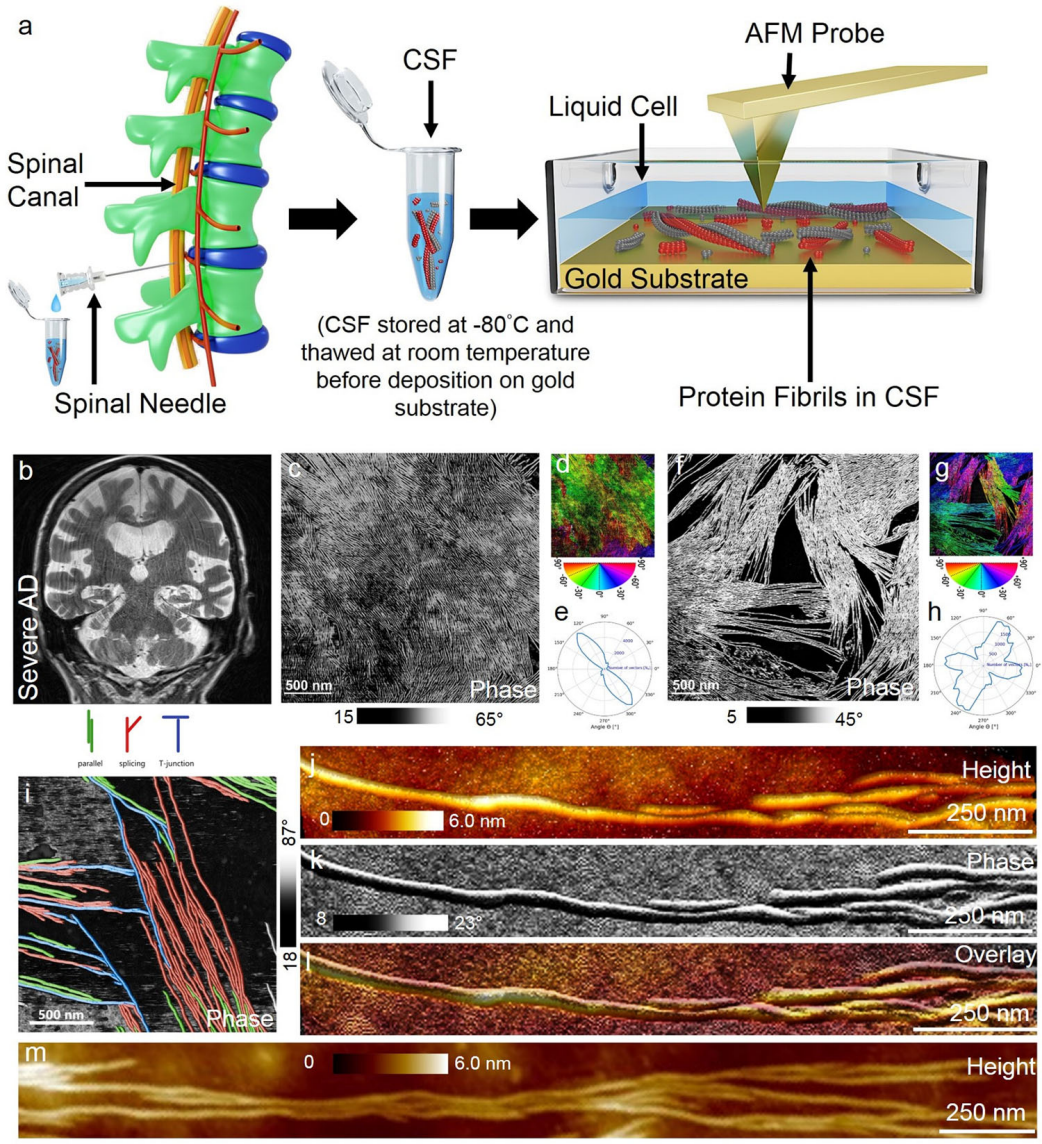
Brain scan and AFM analysis of CSF from an individual with severe AD. **a** Schematic of the CSF extraction and deposition on a gold substrate placed within a liquid cell for AFM imaging under standard laboratory conditions (objects are not shown to scale). **b** A representative T1-weighted coronar MRI scan from patient KSSG:38 showed severe global brain atrophy (global cortical atrophy (GCA) scale 2) with a predominant temporal atrophy pattern (medial temporal atrophy (MTA) index 3-4). **c** Large-area phase-contrast AFM image showing fibrils in CSF from patient KSSG:38 arranged in a 2D format. **d** Colour-coded orientation distribution of the fibrils for the phase-contrast AFM image is shown in **b** and the corresponding polar plot distribution of the angles (**e**). **f** Large-area phase contrast image of fibrillar protein aggregates in a closely packed arrangement and the corresponding distribution in angles is shown in colour-coded phase image (**g**) and polar plot (**h**). **i** Phase-contrast AFM image showing sparsely arranged protein fibrillar aggregates in either parallel (green colour code), spliced (red colour code) or in T-junction (blue colour code) format. **j**–**l** High-resolution AFM height, phase and overlayed images showing well-resolved individual fibrils detected in the CSF from patient KSSG:38. **m** AFM height map of the ultralong single fibrils.

Here, we used liquid-based AFM (see Fig.1a for the schematic of CSF extraction and deposition within the AFM liquid cell) to resolve and quantify differently sized single protein aggregates from oligomers, protofibrils to mature fibrils in CSF extracted from patients presenting with cognitive complaints. CSF of patients with only subjective cognitive decline (SCD, *n* = 7, age = 61.0 ± 8.9 years, MoCA score = 26.7 ± 1.5), mild cognitive impairment (MCI) without specified aetiology (*n* = 1, age = 60.8, MoCA score = 20), MCI due to AD (MCI AD, *n* = 10, age = 69.2 ± 11.0 years, MoCA score = 23.0 ± 3.5), patients with AD dementia (ADD, *n* = 11, age = 72.0 ± 12.7 years, MoCA score = 15.7 ± 6.3), Lewy Body dementia (LBD, *n* = 2, age = 74.0 ± 5.6 years, MoCA score = 10.0) as well a patient with vascular dementia (VDD, *n* = 1, age = 76.4 years, MoCA score = 25.0) were analysed using AFM. The individual patient details are presented in Table 1 and a group-wise summary is given in Supplementary Table S1. Our findings emphasise the importance of recording protein fibril morphology directly in CSF and their implications in better understanding the molecular pathogenesis of AD and identifying probable targets for therapeutic intervention are discussed. In the next sections, we describe the results from AFM based analysis of CSF from representative patients with advanced stage ADD (identification label: KSSG:38 and KSSG: 36), early onset, mild ADD (KSSG: 15), MCI AD (KSSG: 2) and SCD (KSSG: 19) and subsequently detail the results obtained from AFM analysis of CSF from patients with SCD.

**Table 1:** Clinical data, mean fibril height and length values obtained from AFM analysis of CSF of the studied patients.

Patient ID	Age	Diagnosis	Fibril Length in CSF (µm)	Fibril Height in CSF (nm)	Aβ42/40 ratio	p-Tau [ng/l]	t-Tau [ng/l]	MoCA score [0–30]
KSSG-2	70	MCI AD	0.5 ± 0.25	3.1 ± 0.7	0.041	138	850	24
KSSG-15	53	ADD	2.1 ± 0.35	2.9 ± 1.1	0.043	76	545	20
KSSG-19	58	SCD	0.3 ± 0.2	2.7 ± 1.2	0.089	25	146	27
KSSG-20	75	PDD	0.5 ± 0.2	1.5 ± 0.2	0.094	51	357	20
KSSG-31	81	ADD	1.9 ± 0.7	2.6 ± 0.4	0.042	161	846	11
KSSG-36	73	ADD	2.4 ± 0.5	4.3 ± 2	0.039	73	439	7
KSSG-37	61	MCI	0.45 ± 0.1	2.9 ± 0.3	0.1	28	189	20
KSSG-38	90	ADD	2.2 ± 0.75	3 ± 0.4	0.04	58	356	3
KSSG-40	84	ADD	2.25 ± 0.6	3.8 ± 1.7	0.064	64	435	20
KSSG-44	78	LBD	1.2 ± 0.3	2.2 ± 0.5	0.108	66	534	
KSSG-46	76	SCD	0 ± 0	0 ± 0	0.073	37	324	25
KSSG-48	57	ADD	1.7 ± 0.5	4.2 ± 1.3	0.05	160	1077	19
KSSG-51	56	MCI AD	0.8 ± 0.25	2.4 ± 0.6	0.062	26	192	26
KSSG-54	79	MCI AD	1.3 ± 0.2	2.9 ± 0.4	0.038	86	580	21
KSSG-57	53	SCD	0.4 ± 0.2	2.2 ± 0.8	0.122	28	181	26
KSSG-66	76	VDD	0 ± 0	0 ± 0	0.084	47	401	25
KSSG-68	57	ADD	2.3 ± 0.4	4.3 ± 1.8	0.033	91	765	18
KSSG-71	61	MCI AD	0.75 ± 0.4	2.5 ± 0.2	0.039	44	387	30
KSSG-74	60	ADD	2.1 ± 0.4	4.1 ± 0.2	0.058	50	370	13
KSSG-77	71	MCI AD	1.2 ± 0.5	2.8 ± 0.3	0.027	188	1171	21
KSSG-83	77	MCI AD	1.45 ± 0.3	3.1 ± 0.1	0.035	102	801	20
KSSG-84	60	SCD	0.25 ± 0.1	1.5 ± 0.3	0.128	59	440	25
KSSG-86	76	ADD	2.1 ± 0.8	4.1 ± 1.6	0.062	47	866	20
KSSG-89	80	ADD	2.2 ± 0.5	3.5 ± 0.9	0.053	98	559	20
KSSG-90	72	MCI AD	1.7 ± 0.1	2.3 ± 0.6	0.048	88	448	22
KSSG-94	80	ADD	1.95 ± 0.2	3.8 ± 0.5	0.041	114	550	22
KSSG-97	60	SCD	0 ± 0	0 ± 0	0.068	37	174	29
KSSG−108	70	LBD	0.35 ± 0.1	1.9 ± 0.5	0.084	12	146	10
KSSG−114	90	MCI AD	1.5 ± 0.3	2.7 ± 0.4	0.037	108	660	20
KSSG−129	51	SCD	0.3 ± 0.1	1.8 ± 0.5	0.122	31	307	28
KSSG−136	60	MCI AD	0.8 ± 0.2	3 ± 0.2	0.05	96	543	
KSSG−137	56	MCI AD	1.1 ± 0.4	2.8 ± 0.2	0.05	39	272	
KSSG−138	69	SCD	0.3 ± 0.15	1.75 ± 0.4	0.084	36	298	27

In the case of patients, KSSG:46, 66 and 97 no fibrillar aggregates were detected in their CSF. Fibril length and height were set to zero if no fibrils were detected. The fibril morphology detected using AFM and quantified in CSF from patients KSSG:19, 57, 84 and 129 were mainly protofibrillar aggregates with nodular morphology as shown in Fig. 3. The morphology of fibrils detected in CSF using AFM from the rest of the patients is summarised in Supplementary Table S1.

## Results

### Detection of ultralong protein fibrils in CSF of patients with severe AD

First, we studied CSF extracted from a patient with severe AD (identification label: KSSG:38). The details on CSF extraction, storage, and preparation of CSF samples on the gold surface for AFM imaging are provided in the materials and methods section. Patient KSSG:38 (90-year-old, male) was clinically diagnosed with severe AD and classified as A + T + N + (A, amyloid, T, tau, N, neurodegeneration) according to the NIA-AA research framework^44^. An overview of the clinical characteristics of all patients is provided in Table 1. Representative structural magnetic resonance imaging (MRI) scans demonstrating patients’ brain atrophy are provided for illustrative purposes. Figure 1b is a T2- weighted MRI scan in the coronary orientation of patient KSSG:38 showing severe global atrophy with a predominance of the temporal cortices and the hippocampus. Figure 1c is a large-area AFM phase-contrast image of long and close-packed protein fibrils resolved in CSF (air-dried on the gold surface) obtained from patient KSSG:38. The corresponding height map is provided in Supplementary Fig. S1. Figure 1d and e are the qualitative (colour-coded map) and quantitative (polar plot) analyses of the orientation distribution of the fibrils shown in Fig. 1c using fiberapp software^45^. Figure 1f is a phase-contrast AFM image recorded in CSF from the same patient KSSG:38 showing close-packed protein fibrils with varying orientations as shown in the colour-coded map (Fig. 1g) and polar plot with the distribution of orientation angles (Fig. 1h), calculated similarly to the orientation analysis in Fig. 1d and e. Figure 1i is a phase-contrast AFM image showing clearly the local arrangement of the fibrils within a sparse network. In particular, we observed that the fibrils in CSF from patient KSSG:38 were mainly organised either in parallel (colour-coded in green), spliced (colour coded in red) or in a t-junction format (colour-coded in blue) as shown in the colour-coded phase-contrast image (Fig. 1i). Such individual protein fibrils are arranged in a mostly non-overlapping manner as both close-packed and sparse structures were prevalently resolved on the gold surface during our AFM measurements as shown in Fig. 1c, f and i. Previously, a non-overlapping but close-packed arrangement of elongated fibrils has been mainly observed for in vitro prepared Aβ42 fibrils in buffer salt solutions using AFM and this observation was attributed to the surface-independent bidirectional growth model adopted by Aβ42 species^37,46^. Figure 1j, k are high-resolution AFM height and phase-contrast images of a single protein fibril in CSF from patient KSSG:38, respectively. The fibril structure appeared elongated and was devoid of any nodular feature characteristic seen in protofibrillar protein aggregates^37,47^. Overlaying the height with a phase-contrast image of the single protein fibril (Fig. 1l) did not show any signs of a fuzzy coat previously visualised in pathological human Tau fibrils using AFM^48^. Figure 1m is a spatially well-resolved AFM height map showing the full length of highly elongated protein fibrils in CSF from patient KSSG:38. Based on sectional analysis of nearly ~3000 individual protein fibrils resolved in CSF from patient KSSG:38 using AFM (representative images shown in Fig. 1c, f, i, j, m and Supplementary Figs. S1, S2 and S3), we calculate the mean fibril length to be (2.2 ± 0.75) μm and the mean fibril diameter (see Supplementary Fig. S2 for calculation of fibril diameter from measured height) to be (3.0 ± 0.4) nm. Conversely, only a small population of spherical particles with sizes ranging from ~0.5 to 4 nm were detected in CSF from patient KSSG:38 (Supplementary Fig. S3, panel b inset). The length and diameter of the fibrils measured in CSF from patient KSSG:38 are comparable with fibrils detected in CSF of other individuals (patient identification number: KSSG:36) classified as A + T + N + with severe AD dementia (see Supplementary Fig. S4 and S5). Although the length of fibrils in CSF of patient KSSG:38 is longer than the length of protein fibrils purified from AD brain tissue^26^, the fibril diameter measured directly in CSF is significantly lower than the fibril diameter of ~7–18 nm reported using cryo-electron microscopy^26^.

### Resolving protein aggregates in CSF from patients at various stages of AD

**Fig. 2:**
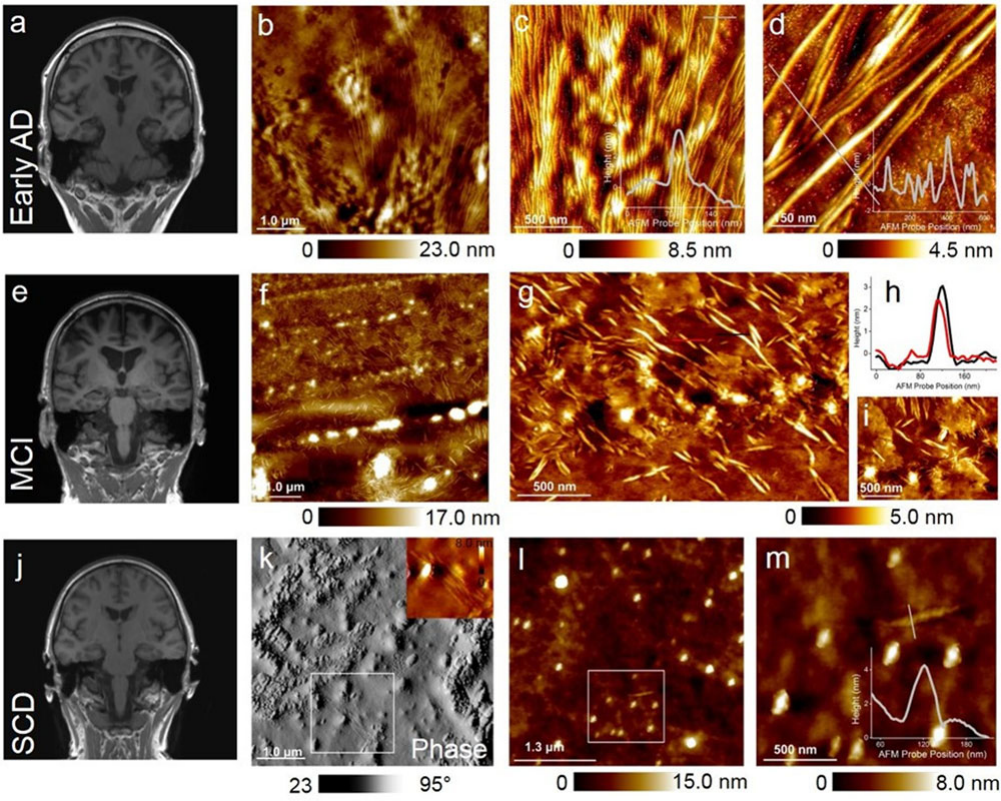
Brain scans and AFM analysis of CSF from individuals with early-onset AD dementia (patient ID: KSSG:15), mild cognitive impairment due to AD (patient ID: KSSG:2) and with SCD and patients with no evidence of AD pathology (patient ID: KSSG:19). **a** Representative T1-weighted coronar MRI scan showing moderate bilateral hippocampal atrophy (MTA 2) of patient KSSG:15 with early-onset AD dementia. **b** AFM height image of sparsely distributed ultralong protein fibrils resolved in CSF of patient KSSG:15. **c**, **d** High-resolution and three-dimensionally rendered AFM height maps of single fibrils resolved within areas in panel **b**. The cross-sectional profile extracted on single fibrils is provided in the bottom right inset of **c** and **d**. **e** Representative T1-weighted coronar MRI brain scan of patient KSSG:2 with MCI due to AD showing moderate global atrophy with a frontal and parietal predominance (GCA 1-2). **f** Large-area AFM height image of fibrillar aggregates present in CSF of a patient KSSG:2. **g** 3-D rendered AFM height image recorded in a region within panel **f** showing clearly shorter and sparsely distributed fibrillar aggregates. **h** Cross-sectional profiles were extracted along the red and black lines indicated in panel **g**. In addition to the short protein fibrils, nanometer sized thin films and spherical particles are also visible in the AFM height images in panels **f**, **g** and **i**. **j** representative T1-weighted coronar MRI brain scan of patient KSSG:19 with SCD with no evidence for AD pathology showing no signs of atrophy. **k** Large-area phase AFM image recorded on CSF from patient KSSG:19. The AFM image reveals mainly spherical particles distributed in the CSF medium. Fibrillar aggregates were also detected as highlighted in the spatially magnified height image (top right inset of **k**) within the white box indicated in **k**. **l** AFM height image was recorded within another region of the same substrate (CSF from patient KSSG:19) showing predominantly spherical aggregates and isolated single fibrils with nodular morphology as indicated in a white box. **m** High-resolution AFM height map recorded within the region indicated by the white box in **l** and the corresponding sectional profile extracted across the single protofibril is shown in the bottom right inset.

Next, we investigated CSF from patients with early-onset, mild AD dementia (KSSG:15, classified as A + T + N + ), with MCI due to AD (KSSG:2, classified as A + T + N + ) and SCD with no signs of AD pathology or neurodegeneration (KSSG:19, classified as A-T-N-) using AFM. Figure 2a is a representative T1-weighted coronar MRI scan of patient KSSG:15 showing moderate bilateral hippocampal atrophy. AFM analysis (Fig. 2b–d) of CSF from patient KSSG:15 revealed the presence of fibrils on the gold surface with a mean length of (2.1 ± 0.35) μm and a mean diameter of (2.9 ± 1.1) nm. Stable population of fibrils were resolved in CSF from patient KSSG:15 with comparable size and morphology to fibrils detected in patients KSSG:38 and KSSG:36 all three patients were classified as A + T + N + . The size of the spherical aggregates detected in CSF from patient KSSG:15 ranged from ~2 to 20 nm. Additonal information on brain imaging and AFM analysis of CSF from patient KSSG:15 is provided in the Supplementary Fig. S6. Figure 2e is a representative T1-weighted coronar MRI brain scan of patient KSSG:2 with MCI due to AD showing moderate global atrophy with frontal and parietal predominance. Interestingly, the AFM images recorded on CSF from patient KSSG:2 (Fig. 2f, g) presented a completely different picture in terms of fibril morphology and the prevalent occurrence of spherical aggregates (size range: ~5–30 nm) compared to AFM analysis of CSF obtained from the patients with late-stage AD dementia (KSSG:38, 36) and early-onset AD dementia (KSSG:15) patients. Based on AFM topographs we calculated a mean fibril length of (0.5 ± 0.25) μm and from the sectional analysis (similar to that shown in Fig. 2h) we calculated a mean diameter of (3.1 ± 0.7) nm for fibrils, which were prevalently detected in CSF from patient KSSG:2. More detailed AFM analysis of fibrils in CSF from patient KSSG:2 is provided in the Supplementary Fig. S7. The size of the spherical aggregates detected in CSF from patient KSSG:2 varied from ~5 to 30 nm. In addition to short fibrils and spherical aggregates (Fig. 2f, g), we also observed thin films in CSF from patient KSSG:2 as shown in the spatially well-resolved AFM topograph (Fig. 2i). The variations in the thickness profile of the nanometer-sized thin films are provided in the Supplementary Fig. S8. Such nanometer-sized thin films classified as nanoplaques have also been reported in the CSF of individuals with AD^49^.

### Characterisation of protofibrillar aggregates in CSF of patients with SCD

**Fig. 3:**
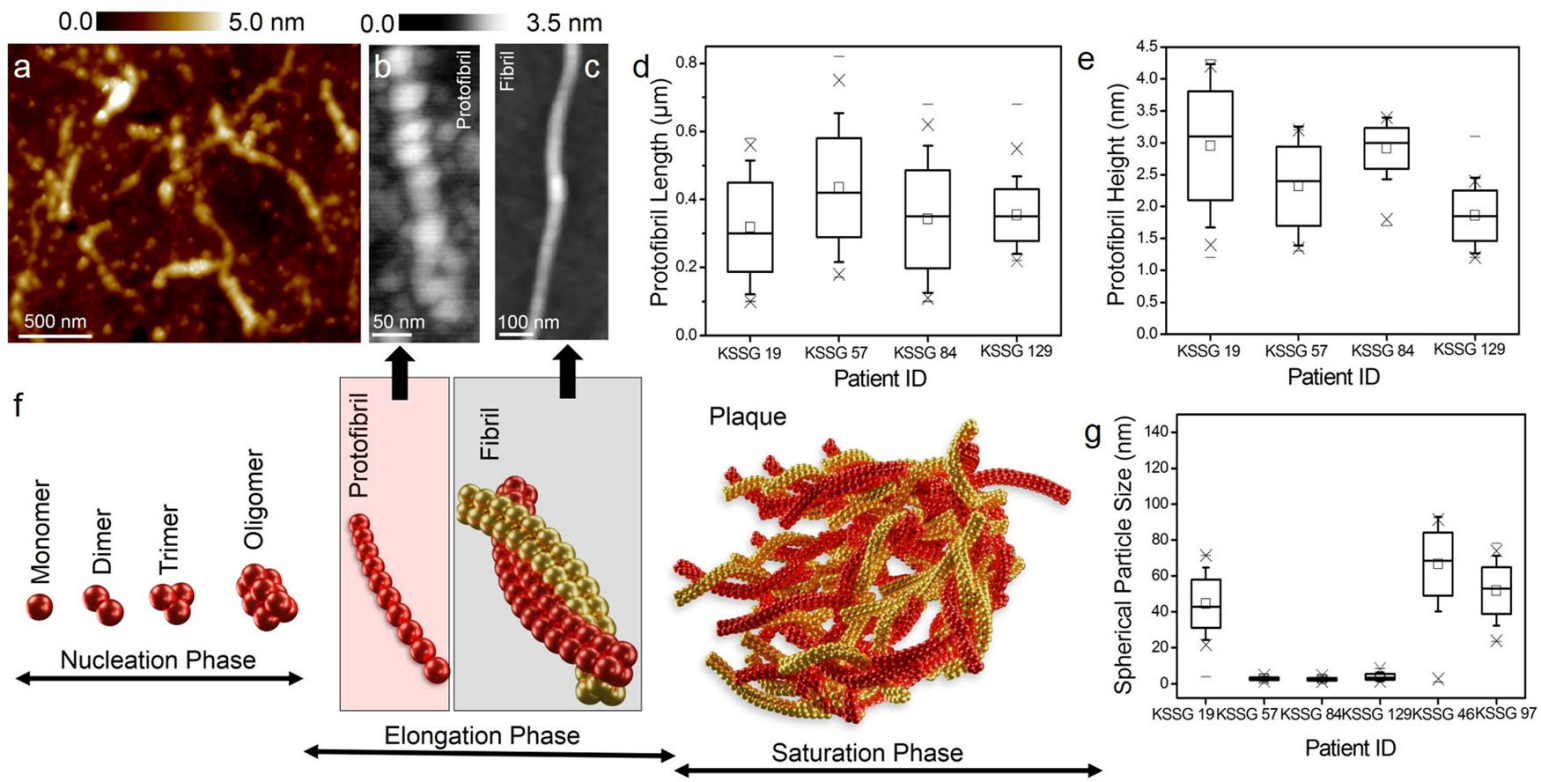
Quantification of protofibrils in CSF from individuals with SCD. **a** Representative AFM image recorded in CSF from patient KSSG:57 (patient with SCD) showing the presence of both spherical and protofibrillar aggregates. **b** AFM topograph of a protofibril resolved in CSF from patient KSSG: 19 (SCD) showing distinct nodular morphology. **c** AFM topograph of a single elongated single fibril resolved in CSF from patient KSSG: 38 (severe AD) showing a smooth surface topology. **d**, **e** Box plots of protofibrillar length and height distribution values obtained from analysis of AFM images recorded in CSF of patients KSSG:19, 57, 84, and 129. Note: No fibrillar aggregates were detected in the CSF of SCD patients KSSG: 46 and 97. **f** Schematic of protein aggregates formed along the assembly pathway of pathological proteins implicated in AD. The red and grey shaded regions indicate protofibril and fibril aggregates. **g** Box plot of spherical particle size distribution in CSF of all SCD patients KSSG:19, 46 57, 84, 97 and 129. The error in the box plots represents the standard deviation of the data sets shown in Fig. 3d, e and g.

Figure 2j is a representative T1-weighted MRI brain scan of the patient with SCD (KSSG:19) showing no signs of atrophy. Analysis of CSF using AFM from the same patient exhibited the presence of mostly spherical aggregates as shown in the phase-contrast AFM image (Fig. 2k) together with short protofibrils. The size of the spherical aggregates in CSF from patient KSSG:19 ranged from ~10–80 nm, based on AFM height images (top inset Fig. 2k) and also see Supplementary Fig. S9. Notably, protofibrils were only scarcely detected in CSF from patient KSSG:19. Based on AFM topographs (Fig. 2l and m) and sectional profiles similar to those shown in the bottom inset Fig. 2m, we calculated a mean length of (0.3 ± 0.2) μm and a mean diameter of (2.7 ± 1.2) nm for fibrils resolved in CSF from patient KSSG:19. To assess whether the presence of ultralong protein fibrils is a general CSF feature or possibly specific to AD, we conducted additional AFM measurements on patients KSSG:19, 46, 57, 84, 97 and 129 who were all classified as SCD (A-T-N-). AFM measurements did not reveal any traces of ultralong protein fibrils in their respective CSF. Yet, AFM analysis of CSF from patients diagnosed with SCD (KSSG:19, 46, 57, 84, 97 and 129) confirmed that only CSF from patients KSSG: 19, 57, 84 and 129 contained protofibrillar aggregates (with nodular morphology) and were present together with spherical particles as shown in Fig. 3a (representative AFM analysis of CSF from patient KSSG:57).

Figure 3b is a high-resolution AFM topograph of a protofibrillar aggregate revealing the nodular morphology detected in CSF of patient KSSG: 57, which is in stark contrast to the morphology of mature fibril (detected in CSF of the patient: KSSG 38, severe AD) shown in Fig. 3c visualised with a smoother surface topology and appears elongated in comparison to protofibrils. The distribution of the protofibril length (Fig. 3d) and height (Fig. 3e) distribution in CSF of patients KSSG 19, 57, 84 and 129 confirms that protein aggregates with heterogeneous size and shape distribution are present in the CSF from SCD patients. Based on the box plot shown in Fig. 3d we calculate the mean length for protofibrils detected in patients KSSG 19, 57, 84 and 129 to be (0.3 ± 0.1) µm, *n*: 78, (0.4 ± 0.1) µm, *n*: 63, (0.34 ± 0.1) µm, *n*: 75 and, (0.35 ± 0.0.7) µm, *n*: 72, respectively. Likewise, the corresponding mean protofibril height was calculated based on the box plot shown in Fig. 3e for patients KSSG 19, 57, 84 and 129 to be (2.95 ± 0.8) nm, (2.3 ± 0.6) nm, (2.9 ± 0.3) nm and (1.85 ± 0.4) nm, respectively.

In addition to protofibrillar aggregates in the CSF of patients with SCD, the presence of spherical particles was also detected during the AFM measurements (see Supplementary Fig. S10 for detailed AFM analysis of CSF from SCD patients). The spherical particles resolved in CSF from patients could also contain protein oligomers, which occur along with protofibrils and mature fibrils that appear as transient aggregates along the assembly pathway of pathological proteins^24,50–53^, as depicted in the schematic Fig. 3f. Oligomeric aggregates of varying size distributions, have been reported previously in CSF of healthy individuals^54^ and those with AD pathology^4,24,31,51,54^. Such protein aggregates represent toxic soluble amyloid-oligomers which could drive neurodegeneration^55^. Based on the box plot shown in Fig. 3g we calculated a mean particle size for all spherical aggregates (*n*: 120 particles for each patient) detected in CSF of patients KSSG 19, 57, 84, 129, 46 and 95 to be (44.5 ± 13.5) nm, (2.8 ± 0.8) nm, (2.5 ± 0.75) nm, (3.8 ± 1.6) nm, (66.6 ± 17.5) nm and (51.8 ± 13.0) nm, respectively.

### Correlating nanoscale imaging data with the clinical diagnosis of the patients

**Fig. 4:**
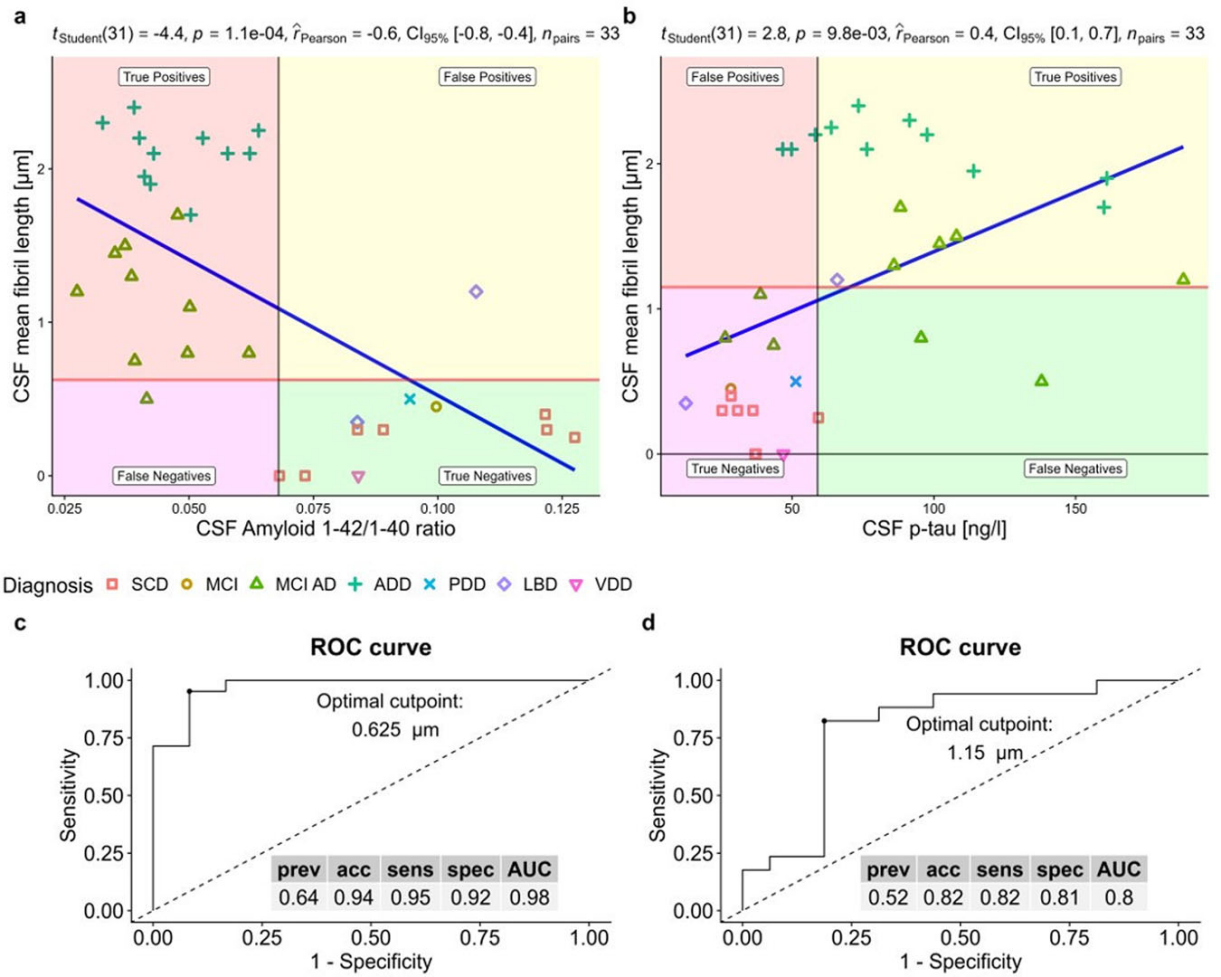
CSF fibril length as a potential biomarker of AD pathology. Scatterplots of the individual mean fibril length in CSF plotted against the CSF Amyloid 42/40 ratio (**a**) and CSF p-tau levels (**b**) as well as the respective Pearson regression results. **c**, **d** Depict the ROC analyses and diagnostic performance measures of CSF fibril length for detecting Amyloid-pathology as determined by a CSF Amyloid 42/40 ratio < 0.068 (**c**), respectively Tau pathology defined as CSF p-tau levels >59 ng/l (**d**). The respective cutoffs were set at the highest Youden-index. The CSF fibril length was significantly, inversely correlated with the CSF Amyloid 42/40 ratio (*r* = −0.62, *p* > 0.001, see **a**) and positively correlated with CSF p-tau (*r* = 0.45, *p* > 0.001, see **b**). When applying a fibril length cutoff of ≥0.625 µm (horizontal red line in **a**) the Amyloid pathology could be determined with an accuracy of 94% (sensitivity: 0.95, specificity: 0.92, see **c**). Regarding Tau-pathology, a cut-off of ≥1.15 µm (highest Youden index) predicted Tau status with an accuracy of 82% (sensitivity: 0.82, specificity: 0.81, see **d**). (prev: prevalence, acc accuracy, sens sensitivity, spec specificity, AUC area under the curve).

**Fig. 5:**
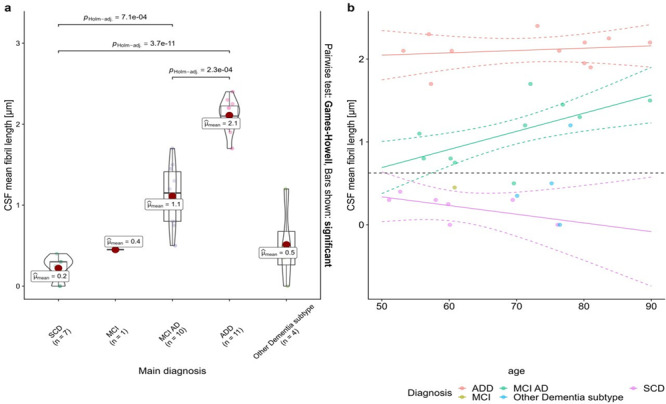
Correlation of fibril length obtained from AFM measurements and CSF with patient diagnostic classification. **a** Combined box and whiskers and violin plots in the style of Tukey of the mean of CSF fibril length per patient group. The mean fibril length was significantly higher in ADD than in MCI and SCD patients as well as higher in MCI AD than SCD patients (Games-Howell pairwise post-hoc tests). **b** CSF fibril length plotted against the age as a function of the group. Regression lines with 95% CI depict the ‘age x diagnosis’ interaction effect on CSF fibril length based on a linear regression model with the predictors’ age, diagnosis and the interaction ‘age x diagnosis’ fitted for patients with either SCD, MCI AD or ADD (*n* = 28).

Differences in protein aggregate morphology recorded in CSF using AFM and the clinical evaluation all patients enroled in this study are detailed in Table 1 and summarized in Supplementary Table S1. Upon comparing the summarized quantitative AFM measurements on fibril length with the clinical evaluation in a patient-specific manner we found that protein fibril length in CSF was inversely correlated with the CSF Aβ42/40 ratio (*r* = −0.6, t(31) = −4.4, *p* < 0.001, see Fig. 4a) and positively correlated with CSF p-tau values (*r* = 0.4, t(31) = 2.8, *p* < 0.01, see Fig. 4b). A fibril length cut-off of ≥0.625 µm determined by the highest Youden-Index could predict the CSF amyloid status of patients with an accuracy of 94% (sensitivity = 0.95, specificity = 0.92, see Fig. 4c) while a cut-off of ≥1.15 µm predicted tau pathology with 82% accuracy (sensitivity = 0.82, specificity = 0.81, see Fig. 4d). The individual mean fibril length in CSF significantly differed between disease groups (one-way ANOVA, F(4) = 48.121, *p* < 0.001, see Fig. 5a). Fibril length was highest in ADD patients (2.1 ± 0.2 µm, see Supplementary Table S1) which were significantly higher than in patients with MCI AD (1.1 ± 0.4 µm, *p*_Holm-adj_ < 0.001) and SCD (0.2 ± 0.2 µm, *p*_Holm-adj_ < 0.001). The difference in fibril length between the MCI AD and SCD groups was also significant (*p*_Holm-adj_ < 0.001). In a linear regression model of fibril length in which age, the disease group and the interaction between age and disease group were the predictors, only the disease group (*F* = 5.8, *p* = 0.009) and the age disease interaction (*F* = 3.5, *p* = 0.047) were significant predictors of CSF fibril length, but not the main effect of age (*F* = 0.9, *p* = 0.343). Simple slope analyses of this linear regression model revealed that age was only significantly correlated with fibril length in patients with MCI AD (*t* = 3.0, *p* = 0.01, see Fig. 5b) but not in patients with SCD (*t* = −0.9, *p* = 0.36) or ADD (*t* = 0.5, *p* = 0.65).

## Discussion

In this study, we demonstrate that liquid-based AFM is well suited to morphologically discern between individual protein aggregates in CSF. Protein fibrils were resolved in CSF and their morphology quantified at a single-fibril level. The ability to clearly quantify protein fibril length and diameter in CSF at physiological conditions validates the usefulness of AFM for monitoring AD onset and progression. Especially, in light of evidence that CSF analysis can be used to detect cerebral Aβ accumulation earlier than PET scans^56^ motivates the need for complementary techniques such as AFM to directly examine the CSF composition. Protein aggregate size, shape profile and structure varied between patients with SCD, MCI AD and ADD. CSF fibril length was observed to be increased in patients with MCI AD and highest in patients with ADD as compared to patients with SCD and non-AD dementia. A surprising result was the presence of a stable and abundant population of micrometre length-sized fibrils detected in CSF from several patients with ADD. These ultralong protein fibrils showed a distinct morphology (diameter: ~3.0 nm, length: few microns) and spatial organisation (the fibrils were closely packed in a mostly non-overlapping format) that was nearly identical across thousands of measured single fibrils. Based on our data and previous reports on the morphology of purified Aβ40^40^, Aβ42^37,46^ and tau^48^ protein fibrils we posit that the observed ultralong protein fibrils detected in CSF from patients with ADD could be attributed to Aβ protein isoform. Although oligomeric and small protofibrillar aggregates have been previously resolved in CSF from MCI and AD patients using AFM^30,31^, the new insights gained from our work for the presence of ultralong fibrils in CSF of ADD patients could trigger further discussion on amyloid clearance mechanisms. Furthermore, the observed increase in fibril length with age in the group of MCI AD patients could indicate a link between fibril growth and AD disease progression. However, fibril length did not change with age in the group with only SCD and manifest ADD suggesting that it is mainly determined by AD pathology and has reached a plateau in patients with manifest dementia. Next, comparing the quantitative morphological data of fibrils in CSF obtained from AFM with biochemical assay based analysis of Aβ and tau protein isoforms in CSF revealed that fibril length parameter was highly correlated with the CSF Aβ42/40 ratio and to a lesser degree with p-tau 181 and predicted amyloid, and to a lesser degree for p-tau pathology, with high accuracy.

Our study has limitations. While we demonstrate the morphological characterisation of protein fibrils in CSF, details on the molecular composition of the fibrillar aggregates detected in CSF remain to be clarified. The chemical nature of the fibrils can be obtained by combing AFM with fluorescence microscopy, which requires the usage of specific fluorophores that can selectively bind to either Aβ or tau protein isoforms in CSF. An alternative strategy to chemically identify protein aggregates in CSF could be to integrate AFM with Raman/IR spectroscopy and then to compare the morphology and spectrochemical signature of the protein aggregates in CSF (unlabelled data) with synthetically purified and prepared amyloid and tau isoforms (labelled data) deposited on a solid substrate. The labelled datasets can then be used as a training layer in a convolutional neural network scheme to classify the unlabelled datasets into either Aβ or tau protein isoforms. In addition to the above-outlined experimental approaches, future efforts will also require larger sample sizes in order to determine whether fibril formation in CSF can also be found in other neurodegenerative diseases or is only specific to AD. We anticipate that a longitudinal follow-up of the patient could further validate whether CSF fibril length can be an indicator for predicting future cognitive decline in patients with MCI AD.

## Materials and methods

### Patients

In total, 238 patients who presented for an initial or follow-up assessment of cognitive complaints at the memory clinic of the Department of Neurology at the Cantonal Hospital St. Gallen were enroled in a pilot study on AFM imaging of blood and CSF in neurodegenerative diseases. For the presented analyses, we initially selected nine patients with either AD dementia (*n* = 3), mild cognitive impairment due to AD (*n* = 1) and patients with only subjective cognitive decline without amyloid pathology (SCD, *n* = 5) from the well-characterized cohort described in our previous publication^35^. The nine patients were selected as they span the AD continuum from patients with only subjective cognitive complaints without biomarker evidence for amyloid-, tau-pathology or neurodegeneration (A-T-N-) to severe dementia due to AD (A + T + N + ) according to the NIA-AA research framework^44^. In a second step, an additional 24 patients were randomly selected from the overall cohort by P.N.N with the clinical information unbeknownst to them, resulting in an analysed total cohort of 33 patients. All participants gave informed consent to participate in the pilot study. When there were doubts concerning patient’s ability to give informed consent, we additionally obtained the informed consent of the relative accompanying the patient. The experimental procedures were approved by the ethics committee of East Switzerland (Number: 202000558) and were performed following the Declaration of Helsinki. All patients received a comprehensive diagnostic workup comprising a neurological and neuropsychological examination, structural MRI imaging and CSF analyses. LUMIPULSE G assays (Fujirebio, Tokyo, Japan) were used to measure hTau Ag, pTau 181, β-amyloid 1–42, β-amyloid 1–40 and the corresponding Aβ 1–42/Aβ 1–40 ratio. Standard laboratory cutoffs were used to define a positive amyloid status (CSF-β-Amyloid 1–42/1–40 ratio < 0.068) and tau status (pCSF p-tau >59 ng/l), whereas atrophy in structural MRI imaging, hypometabolism in FDG-PET imaging or elevated total tau levels (>410 ng/l) were regarded as evidence of neurodegeneration. Clinical diagnoses were reached in an interdisciplinary consensus. Overall, seven patients presented with solely subjective cognitive decline (SCD) and one patient had mild cognitive impairment of unspecified aetiology. Ten were diagnosed with mild cognitive impairment due to AD (MCI AD) and eleven patients with probable AD dementia with biomarker evidence (ADD) according to the NIA-AA criteria of 2011^57^. One patient was diagnosed with Parkinson’s disease dementia (PDD), two with Lewy Body dementia (LBD)^58^ and one patient with dementia due to vascular disease (VDD)^59^ according to the respective diagnostic criteria.

### Preparation of CSF samples for AFM analysis

Directly after the lumbar puncture, CSF samples were stored at −80 °C at the laboratory, Zentrum für Labormedizin St. Gallen. Following the CSF retrieval, the samples were thawed under standard laboratory conditions to a liquified state before deposition on gold thin films. The gold thin films (~100 nm of Au (111) on mica substrate) were commercially purchased from Phasis, Inc. Before deposition of CSF, the as-received gold substrates were thoroughly cleaned (acetone rinsing followed isopropyl alcohol rinsing and blow drying with N_2_). Only atomically flat samples (verified using scanning tunnelling microscope, Pt/Ir tip, E-scanner, constant current mode, multimode-Bruker 8) with an overall surface roughness was ≤0.5 nm over an area of 5 µm^2^ (verified using AFM, tapping mode, multimode-Bruker 8) were used for CSF deposition. Gold thin films with surface defects^60^ were also observed in some of gold samples as-received from Phasis Inc and such samples were excluded from usage for protein imaging experiments.

### AFM measurements

AFM measurements were conducted in tapping mode using a multimode 8 Bruker instrument equipped with an E-scanner. For the AFM tip, a SCOUT 150 HAR silicon AFM tips with a high aspect ratio was used in tapping mode (gold reflective backside coating, force constant 2 N/m, resonant frequency: 150 kHz, NuNano). Before inserting the AFM tip in the holder, the AFM tip was cleaned by rinsing in acetone for 30 s followed by rinsing in isopropyl alcohol for ~1 min followed by blow-drying with compressed air. AFM measurements were conducted directly in the CSF medium (without air-drying process) by injecting ~10 µL of CSF into the closed type liquid-cell holder placed over the gold substrate. For the AFM measurements at the liquid-solid interface, the AFM tip and cantilever were immersed in CSF and the resonant frequency of the tip was reduced by ~30%, due to the damping of cantilever dynamics. AFM measurements were also conducted on air-dried samples by first depositing ~10 µL of CSF on the clean gold thin films followed by drying in the air (~2 h) and then placing the air-dried CSF on gold disks on top of the E-scanner for AFM imaging. No differences in fibril length or prevalence were detected for CSF samples that were air-dried and when the same CSF was analysed under hydrated conditions. In particular, we monitored the shrinking factor by directly comparing the fibril height measured in the CSF medium retained in a hydrated condition and the same CSF samples analysed upon air drying. From such comparative analysis, we derived a shrinking factor of 0.8 ± 0.1. The AFM images were analysed using Nanoscope software (Bruker), subjected to flattening for extraction of surface roughness values of gold thin films, and height and length analysis of the protein fibrils. The orientation map of ultralong fibrils in CSF shown in Fig. 1d, e, g and h was analysed using Fiberapp, open-source software^45^.

### Statistics and reproducibility

Statistical analyses were performed in the R environment for statistical computing (version 4.2.0, R Core Team (Vienna, Austria; 2018), R Project for Statistical Computing). To compare the clinical characteristic between disease groups exploratory analyses of variance (ANOVA) tests for numerical data and chi-square tests for categorical data were conducted as implemented in the ‘arsenal’ package^61^ (see Supplementary Table S1). Inferential statistics were performed for the main AFM measure individual mean CSF fibril length as fibrils were the most prevalent protein aggregate species in our patients. Of note, no fibrils were detected in three patients (KSSG: 46—SCD, KSSG: 66—VDD, and KSSG- 97—SCD, see Table 1). We based the fibril length as zero for these patients. Simple regression analyses between the dependent variable CSF fibril length and the CSF Aβ 42/40 ratio, respectively the p-tau levels were performed and plotted using the ‘ggstatsplot’ package^62^. Moreover, pairwise disease group comparisons were performed with Games-Howell tests with Holm-Bonferroni adjustments applied for multiple testing. Moreover, we determined the accuracy, sensitivity and specificity of CSF fibril length as a predictor of CSF amyloid status as determined by the Aβ 42/40 ratio and the CSF p-tau status by calculating receiver operating characteristics (ROC) for cutpoints which yielded the highest Youden-index (sensitivity + specificity -1) using the ‘cutpointr’ package^63^. A linear regression model of CSF fibrillar length with the predictors’ age, disease and the age x disease interaction was fitted and the model estimate was tested with an ANOVA. Simple age regression slopes were analysed for each disease group using the ‘interaction’ package.

### Reporting summary

Further information on research design is available in the Nature Portfolio Reporting Summary linked to this article.

## Supplementary information

Peer Review File

Supplementary Information

Description of Additional Supplementary Files

Supplementary Data 1

Reporting Summary

## Data Availability

All data needed to evaluate the conclusions in the paper are present in the paper and/or the Supplementary Materials. Additional data related to this paper may be requested from the authors. The source data for the statistical plots shown in Figs. 3, 4 and 5 are also provided as Supplementary Data 1 file.
