# Supplementary material for: Protein fibril length in cerebrospinal fluid is increased in Alzheimer’s disease
Source: Commun Biol. 2023 Mar 8;6:251. doi: 10.1038/s42003-023-04606-7 (PMC9995532; doi:10.1038/s42003-023-04606-7)
Supplement: Supplementary file 2 — Supplementary Information [file 42003_2023_4606_MOESM2_ESM.pdf]

Protein fibril length in cerebrospinal fluid is increased in Alzheimer's disease

Peter Niraj Nirmalraj[1*], Thomas Schneider[2], Lars Lüder[1] and Ansgar Felbecker[2*]

1. Transport at Nanoscale Interfaces Laboratory, Swiss Federal Laboratories for Materials Science and Technology, Dübendorf, CH-8600, Switzerland. 2. Department of Neurology, Cantonal Hospital St. Gallen, St. Gallen, CH-9007, Switzerland.

Contents

Table S1: Summary of clinical and AFM data of patients by diagnosis group

Fig. S1: Brain imaging and CSF analysis from a patient with severe AD dementia (KSSG:38).

Fig. S2: Structural analysis of fibrils from patient KSSG:38.

Fig. S3: Statistical analysis of fibril length distribution from patient KSSG:38.

Fig. S4: Brain imaging and CSF analysis from patient KSSG:36 with mild AD dementia.

Fig. S5: Large-area AFM analysis of CSF from patient KSSG:36.

Fig. S6: Brain imaging and CSF analysis from a patient with early-onset AD dementia (KSSG:15).

Fig. S7: Brain imaging and CSF analysis from a patient with MCI due to AD (KSSG:2).

Fig. S8: Morphological analysis of plaques in CSF from patient KSSG:2 with MCI.

Fig. S9: Brain imaging and CSF analysis from a patient with SCD (KSSG:19).

Fig S10: Morphological analysis of CSF from individuals with SCD (no AD pathology).

Table S1: Summary of clinical and AFM data of patients by diagnosis group. The number of observations, mean and standard deviation (in parentheses) per diagnosis group as well as the p-values of between-groups differences based on ANOVA tests for numerical data and on chi-square tests for categorical data are given.

	SCD (N=7)	MCI (N=1)	MCI AD (N=10)	ADD (N=11)	PDD (N=1)	LBD (N=2)	VDD (N=1)	Total (N=33)	p value
**Gender**	4 (57.1%)	0 (0.0%)	6 (60.0%)	4 (36.4%)	1 (100.0%)	2 (100.0%)	1 (100.0%)	18 (54.5%)	0.408[1]
**Age**	61.04 (8.92)	60.80 (NA)	69.18 (11.08)	72.00 (12.71)	75.20 (NA)	74.04 (5.60)	76.42 (NA)	68.84 (11.15)	0.440[2]
**Adjusted MoCA score [/30]**	26.71 (1.50)	20.00 (NA)	N = 8 23.00 (3.51)	15.73 (6.29)	20.00 (NA)	N = 1 10.00 (NA)	25.00 (NA)	N = 30 20.63 (6.42)	0.001[2]
**MMSE score [/30]**	N = 1 28.00 (NA)	NA	N = 1 0.00 (NA)	N = 1 0.00 (NA)	NA	NA	NA	9.33 (16.17)	NaN[2]
**Clock Test [/7]**	N = 3 7.00 (0.00)	N = 1 4.00(NA)	N = 2 6.00 (1.41)	N = 8 4.12 (2.17)	N = 1 7.00 (NA)	N = 1 2.00 (NA)	N = 0 NA	N = 16 4.94 (2.14)	0.172[2]
**CSF Amyloid 1-42/1-40 ratio**	0.10 (0.03)	0.10 (NA)	0.04 (0.01)	0.05 (0.01)	0.09 (NA)	0.10 (0.02)	0.08 (NA)	0.06 (0.03)	< 0.001[2]
**CSF p-tau [ng/l]**	36.33 (11.12)	28.40 (NA)	91.43 (48.57)	90.21 (40.28)	51.30 (NA)	39.20 (37.76)	46.90 (NA)	71.69 (43.68)	0.061[2]
**CSF t-tau [ng/l]**	267.14 (105.22)	189.00 (NA)	590.40 (293.97)	618.91 (235.56)	357.00 (NA)	340.00 (274.36)	401.00 (NA)	491.18 (266.15)	0.062[2]
**CSF fibril length [µm]**	0.22 (0.16)	0.45 (NA)	1.11 (0.39)	2.11 (0.20)	0.50 (NA)	0.77 (0.60)	0.00 (NA)	1.16 (0.81)	< 0.001[2]
**CSF fibril height [nm]**	1.42 (1.04)	2.90 (NA)	2.76 (0.28)	3.69 (0.61)	1.50 (NA)	2.05 (0.21)	0.00 (NA)	2.63 (1.16)	< 0.001[2]

1. Pearson's Chi-squared test
2. Linear Model ANOVA

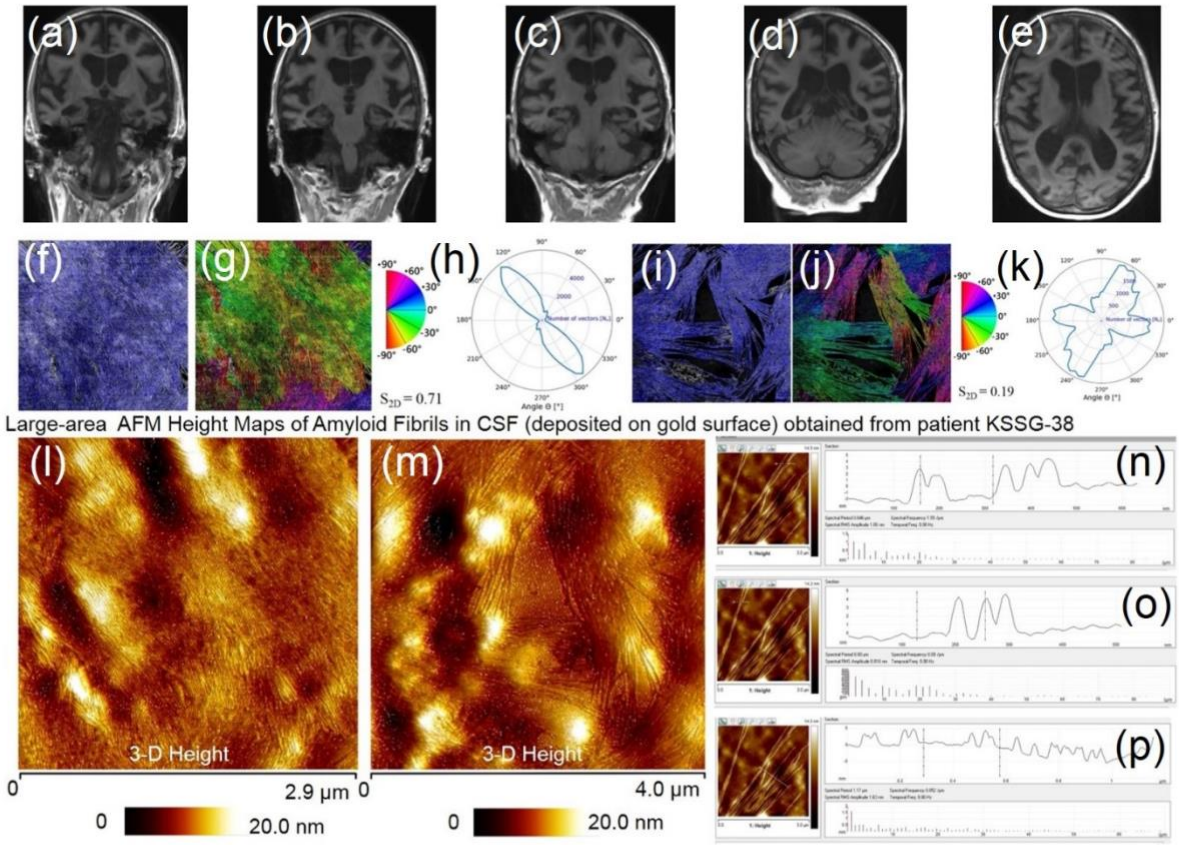

Fig. S1: Brain imaging and CSF analysis from a patient with severe AD dementia (KSSG:38). **(a-e)** Selected coronary and axial MRI brain scans showed advanced global brain atrophy (GCA 2) with a predominant temporal atrophy pattern (MTA 3-4). **(f)** Large-area AFM phase-contrast image colour coded using ImageJ software (1735 individual fibrils detected). The phase-contrast image without the colour coding is shown in **Fig. 1b** (main manuscript). **(g)** Colour-coded AFM image showing the orientation angles of the fibrils (fiberapp software[1]) and the corresponding polar plot **(h)** showing the fibril orientation within the AFM image. The fibrils are densely packed in a mostly non-overlapping manner. The fibrils are tracked individually to measure the length distribution. **(i)** Large-area phase contrast image of fibrillar protein aggregates in a closely packed arrangement and the corresponding distribution in angles is shown in colour-coded phase image **(j)** and polar plot **(k)**. In the colour-coded AFM image shown in panel **i**, nearly 2057 individual fibrils were tracked using ImageJ software. (**l** and **m**) High-resolution 3-D AFM height image of fibrils (corresponding height image for the phase-contrast data shown in panels **g** and **j**. (**n-p**) AFM height and corresponding sectional profile analysis extracted along the lines indicated in the AFM height image. The sectional profiles show the differences in height of the individual fibrils detected using AFM. AFM images shown in panels in **Fig. S1** were recorded on CSF from patient KSSG:38 and deposited on clean gold surfaces followed by air-drying before AFM imaging in tapping mode.

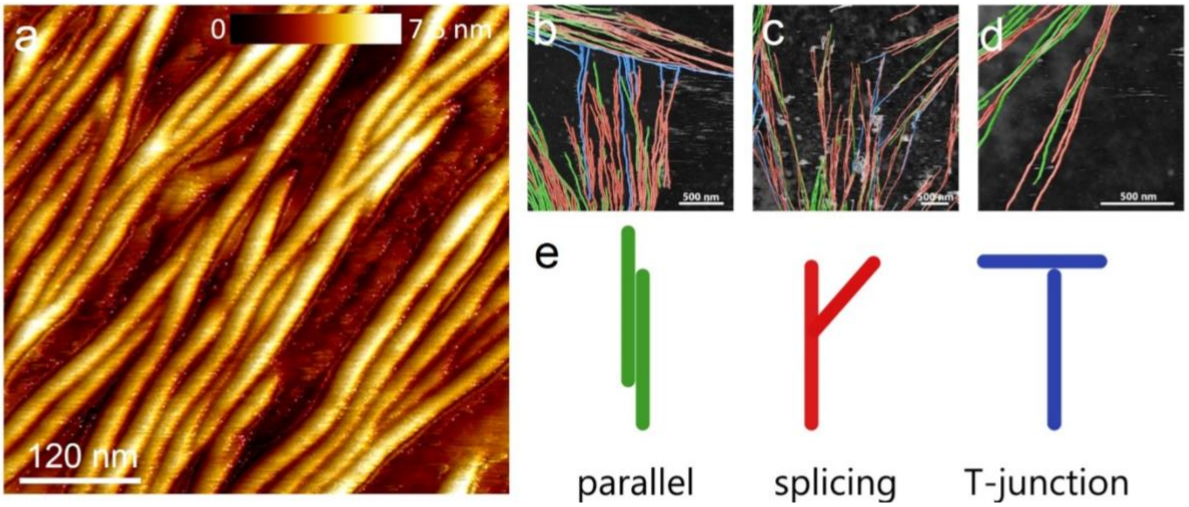

Fig. S2: Structural analysis of fibrils from patient KSSG:38. (a) High-resolution AFM height image of single fibrils resolved within a sparse fibrillar network area of CSF from patient KSSG:38. The fibrils appear with smooth surface morphology suggestive of mature and elongated fibrils compared to nodular morphology only observed for protofibrils. The full length of the single fibrils is not seen from this spatially magnified AFM image. **(b-d)** AFM height images (in grayscale) and colour-coded according to their morphological arrangement of the fibrils present with a sparse network. The fibrils as seen from the AFM images are arranged as either oriented in parallel configuration (coded in green), spliced (coded in red) or in a T-junction (coded in blue) format. The height of an individual fibril is calculated by measuring the height at the middle section of the fibril compared to the height of the underlying surface to get an accurate height value from the AFM height images. The observed width of the fibril from the AFM image is a convolution between the AFM tip and fibril width and is larger than the true width of the fibril. As the measured fibril height does not depend on the geometry of the AFM tip and as the height equals the diameter of a cylinder, it is possible to estimate the single fibril diameter from only AFM height data. AFM image shown in **panel a** was recorded in hydrated CSF medium drop cast on Au(111) and imaged immediately upon CSF deposition.

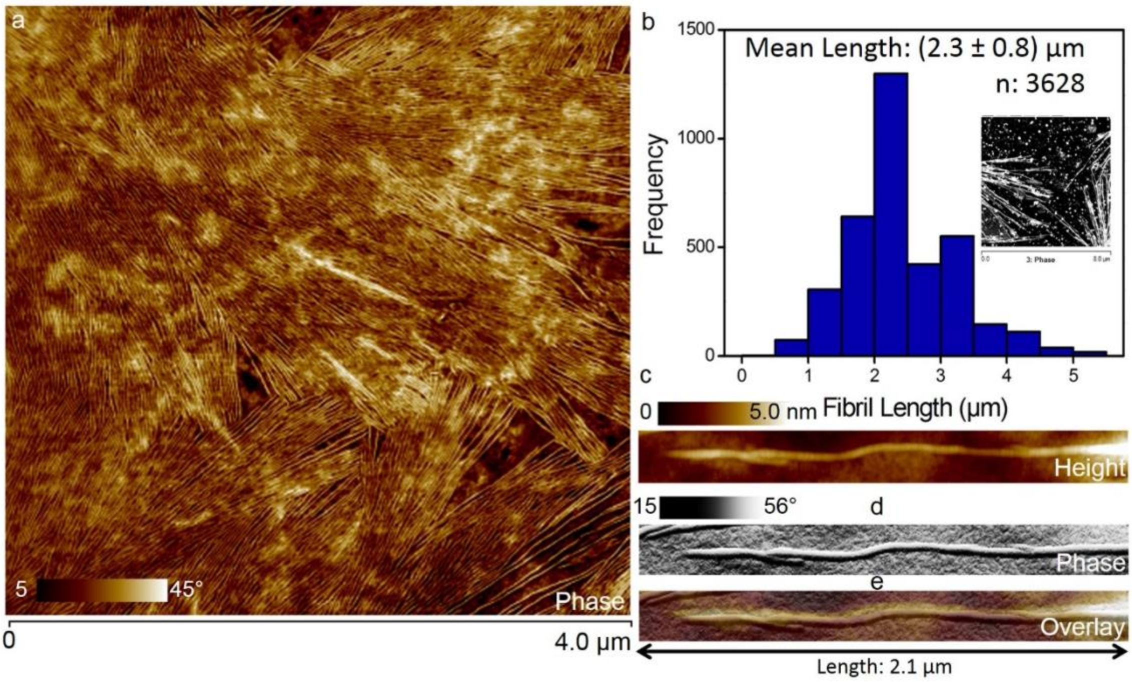

Fig. S3: Quantitative analysis of fibril length distribution from patient KSSG:38. (a) Large-area AFM phase image showing the dense and non-overlapping arrangement of fibrils in CSF from patient KSSG:38. **(b)** Based on the AFM height and phase images acquired after analysing the CSF from patient KSSG:38, we calculate a mean fibril length of (2.3 ± 0.8) µm as shown in the blue histogram. The inset in panel **b** is a phase-contrast AFM recorded on other areas of the same sample where the image from panel **a** was also recorded showing that ultralong fibrils were prevalently detected on the gold surface with variations in surface coverage (sparse or dense network). The height profile **(c)**, corresponding phase-contrast **(d)** and overlay of height and phase-contrast data **(e)** recorded on a single fibril show the fibril morphology with high-spatial clarity. Our AFM measurements did not reveal any fuzzy coat surrounding the fibril as previously reported using AFM exclusively for pathological human tau fibrillar aggregates[2].

In addition to the mature and elongated fibrils prevalently detected in CSF from patient KSSG:38, a small population of spherical particles was also observed from the AFM measurements (right inset panel **b**, S3). Based on the AFM topography images and size profile measurements we estimated the size of the spherical particles to range from ~0.5 to 4.0 nm.

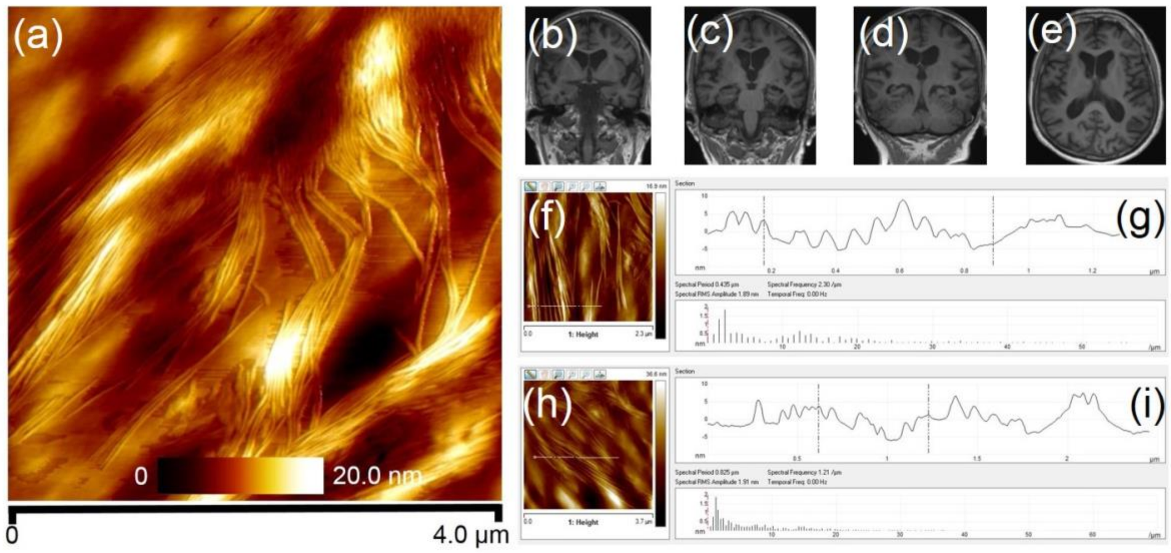

Fig. S4: Brain imaging and CSF analysis from patient KSSG:36 with severe AD dementia. MRI imaging and CSF analysis from a patient with mild AD dementia (KSSG:36). **(a)** Large-area AFM image of fibrils resolved in CSF from KSSG:36. **(b-e)** T1 weighted coronar **(a-c)** and axial **(d)** MRI brain scans showing advanced global brain atrophy (GCA 2) with a predominant temporal atrophy pattern (MTA 3) typical for AD. **(f-g)** AFM image and sectional analysis extracted along the AFM topograph showing the differences in fibril height measured using AFM in tapping mode. **(h-i)** AFM image and sectional analysis extracted along another region in an AFM image recorded in CSF from patient KSSG:36. The local corrugations and differences in fibril height and length are visible from the AFM data. Based on sectional analysis of numerous such fibrils resolved from AFM measurements, we calculated a mean fibril length of (2.25 ± 0.58) μm and a mean fibril diameter of (4.3 ± 1.8) nm.

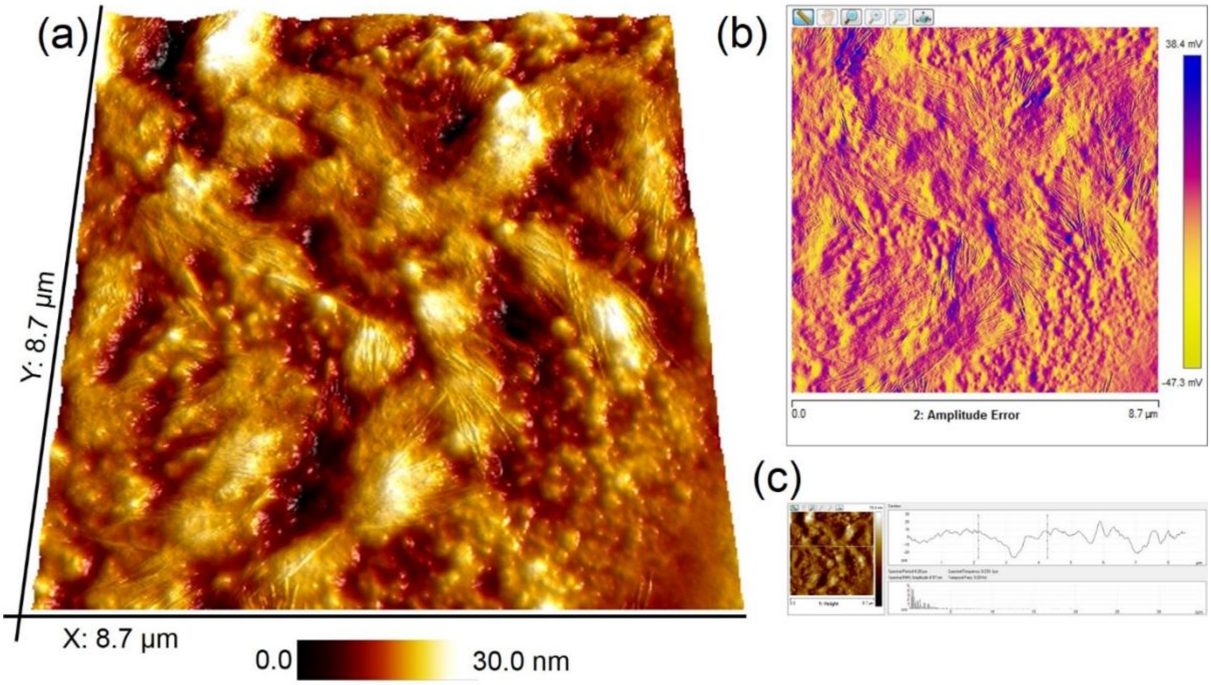

Fig. S5: Large-area AFM analysis of CSF from patient KSSG:36. (a-b) Large-area AFM height and amplitude error image recorded in CSF from patient KSSG:36 (mild AD dementia) showing the presence of both long fibrils and spherical particles. The AFM measurements were conducted on the same sample on which **Figs. S4** was recorded (showing mostly fibrillar aggregates), highlighting that the sample topography varies by location on the Au (111) substrate. For this reason, the gold substrates with both air-dried and CSF in a hydrated state are thoroughly analysed by acquiring nearly 50 images at numerous locations on the same sample to generate a complete picture of the variations in fibril morphology and heterogeneity in composition between the various polymorphs of the pathological protein aggregates. **(c)** Sectional analysis extracted along the line indicated in the AFM image showing the differences in height profile between fibrillar and spherical aggregates detected in the AFM topography. AFM image shown in **panel a** was recorded in CSF medium retained in a hydrated state. In addition to the fibrillar aggregates detected in CSF from patient KSSG:36, a significant population of spherical aggregates with sizes ranging from ~2 to 10 nm were also detected from the AFM measurements.

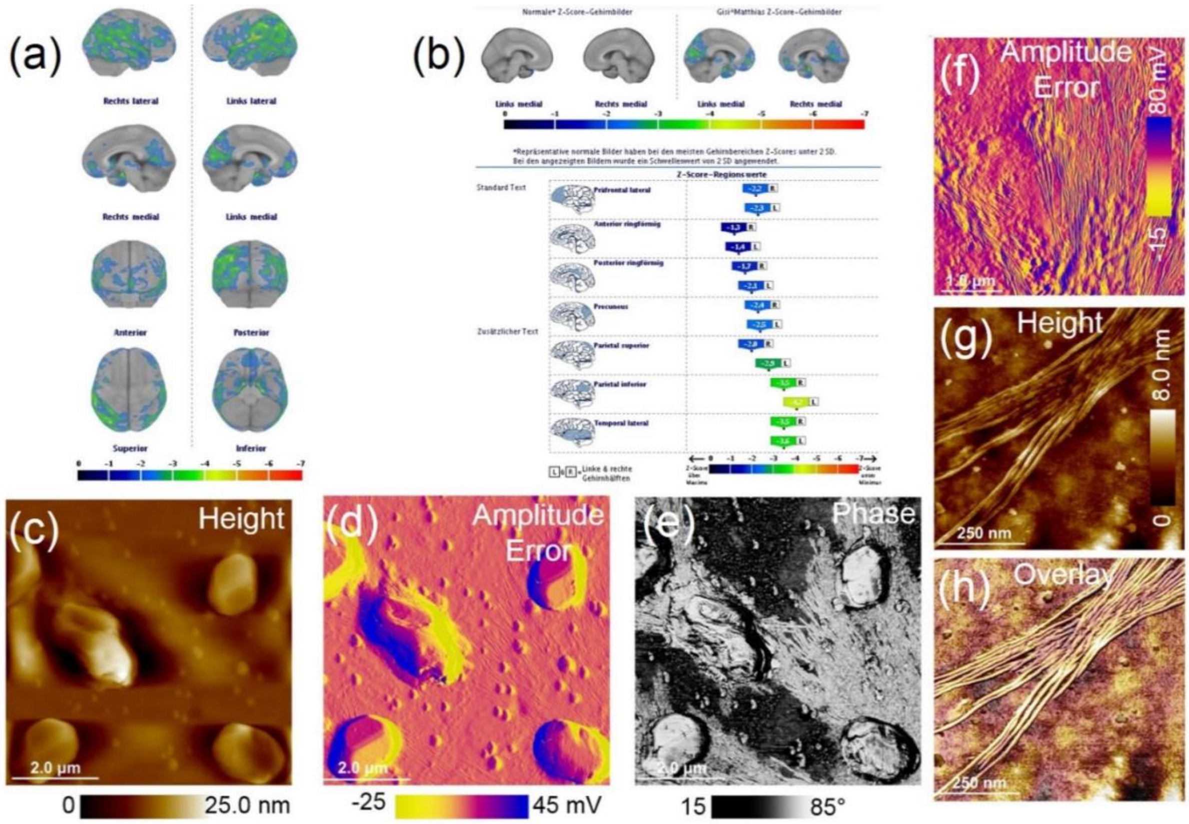

Fig. S6: FDG-PET CT imaging and CSF analysis from a patient with early-onset AD dementia (KSSG:15). **(a)** and **(b)** FDG-PET analysis showing a bitemporal and biparietal hypometabolism supporting the diagnosis of AD dementia. Height **(c)**, amplitude error **(d)** and phase-contrast data **(e)** were recorded in CSF drop cast and dried on a gold surface. The AFM datasets recorded simultaneously highlight the importance of optimising phase-contrast imaging to resolve low-lying fibrillar aggregates present within large particles as seen in panel **c** (height image). Often, relying only on height data to study samples such as CSF, which has several elements of varying size, shape and morphologies could lead to missing important information such as the presence of fibrillar aggregates (~1-5 nm in height) present within larger structures (visible in AFM images, S6, panel **c**). Phase-contrast information is based on the surface properties such as viscoelastic dissipation, adhesion and friction, which records the lag in phase between the cantilever oscillation relative to the response of the cantilever[3]. For high-resolution phase-contrast images, the drive amplitude is reduced as the free amplitude goes lower than the setpoint and then the setpoint value is adjusted accordingly for better tracking of fibril morphology. The phase-contrast images recorded during our AFM measurements were obtained in both the net attractive (phase larger than 90°, softer tapping) and repulsive phase regime (phase lower than 90°, harder tapping). **(f)** Large-area AFM image (amplitude error) of fibrils resolved in between the large particles shown in panel **c**. The amplitude error is additional information that provides more details on both the surface corrugation and overlying fibrils. **(g-h)** Height and height with phase-contrast image overlay of fibrils closely packed in spliced and parallel oriented fibrils on gold surface. Based on AFM

sectional analysis of single fibrils detected in CSF from patient KSSG:15 we calculated a mean fibril length of (2.1 ± 0.35) µm and a mean fibril height of (4.1 ± 1.8) nm. The size of the spherical aggregates ranged from ~2 to 20 nm.

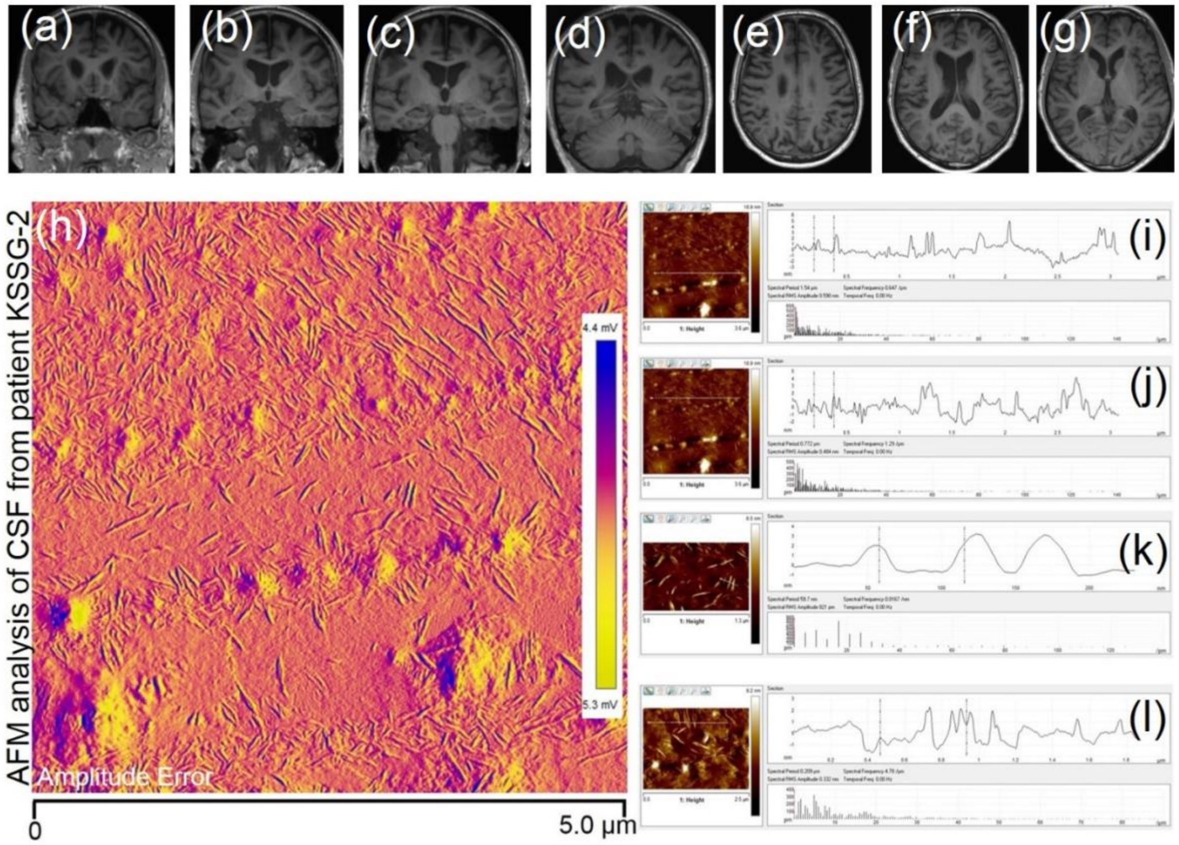

Fig. S7: Brain imaging and CSF analysis from a patient with MCI due to AD (KSSG:2). **(a-g)** T1-weighted coronar and axial MRI brain scans showing mild global atrophy (GCA 1-2) with frontal and parietal predominance. **(h)** Large-area AFM scan (amplitude error) of CSF deposited and air-dried on gold surface. The AFM image reveals fibrils, which are visibly shorter compared to the fibrils detected in patient KSSG:38 with severe AD (Fig. S1.S2 and S3). Based on AFM sectional analysis as shown in panels **i-l** on single fibrils detected in CSF from patient KSSG:2 we calculated a mean fibril length of (0.45 ± 0.22) µm and a mean fibril height of (3.1 ± 0.7) nm. The size of the spherical aggregates detected in CSF from patient KSSG:2 varied from ~5 to 30 nm.

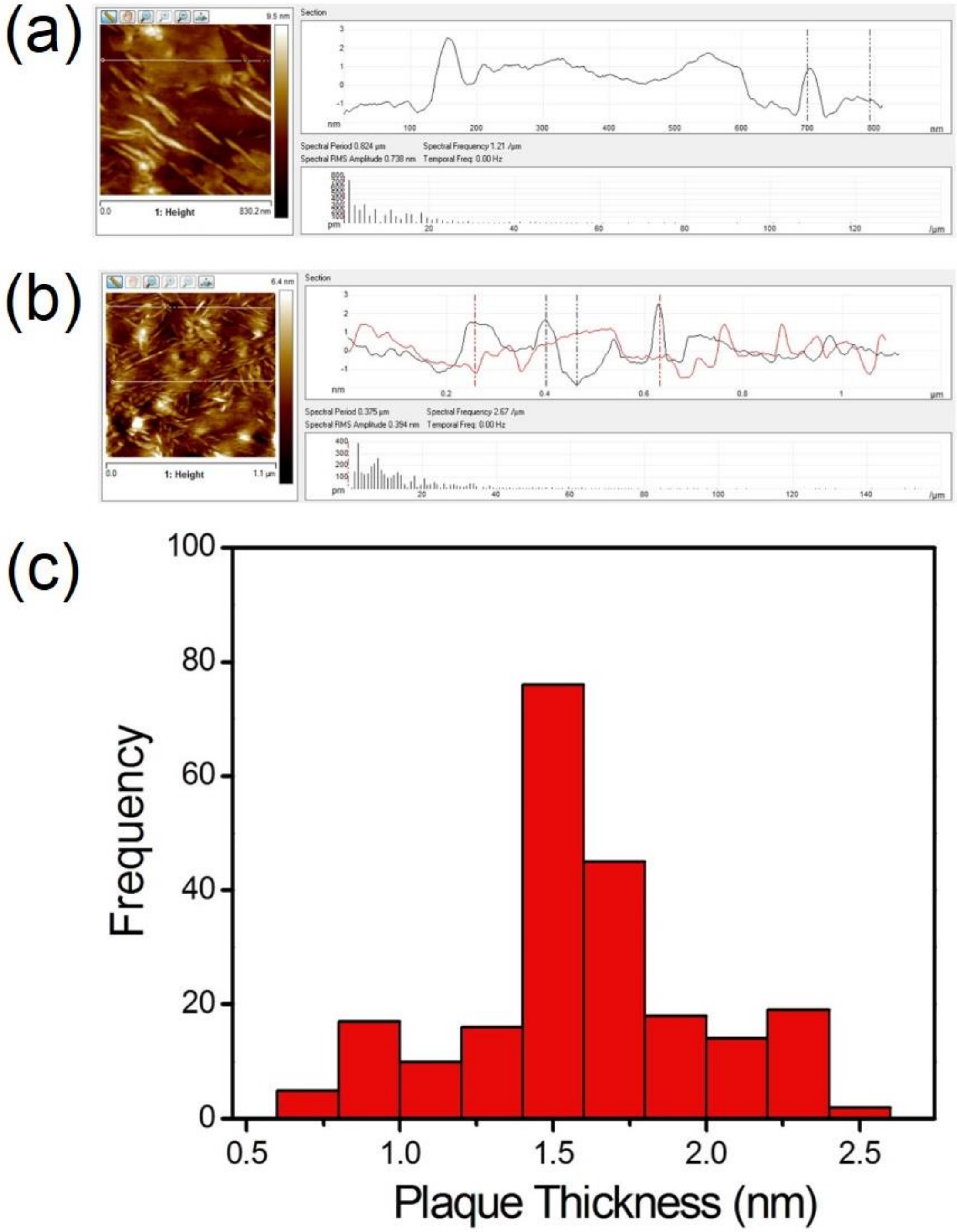

Fig. S8: Morphological analysis of plaques (nanometer-sized thin films) in CSF from patient KSSG:2 with MCI. (a-b) AFM height maps of the plaques and the corresponding line sectional analysis to extract the height profile of the plaques present within air-dried CSF samples on a flat gold surface. **(c)** Based on the AFM analysis of the thickness of the plaques (n.: 222) we calculated a mean plaque thickness of (1.6 ± 0.35) nm. Such few nanometer thick plaques were only observed in CSF for patient KSSG:2 and were neither observed in CSF from patients with severe AD (KSSG:28, 36) nor in patients with early-onset AD (KSSG:15). The clinical evaluation of all the patients is provided in table

1 in the manuscript. AFM images shown in panels **a** and **b** were recorded in CSF retained in a hydrated state.

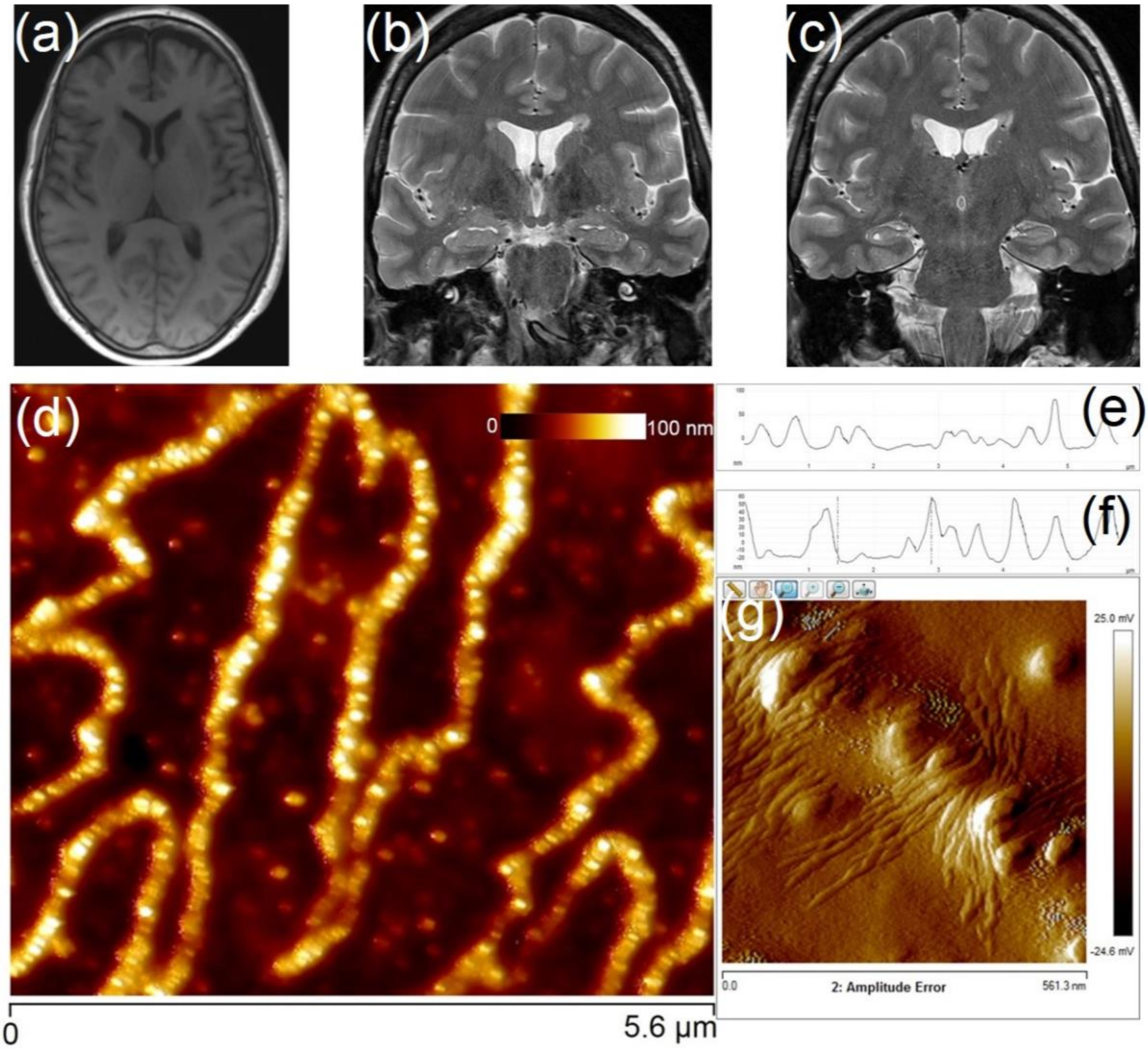

Fig. S9: Brain imaging and CSF analysis from a patient with SCD (KSSG:19). **(a)** T1-weighted axial and T2-weighted coronar. **(b-c)** MRI brain scans show a normal brain volume on visual inspection and volumetric analysis. **(d)** Large-area AFM image showing mostly spherical particles of varying sizes arranged in a linear format. **(e-f)** Cross-sectional profiles extracted across different regions of the AFM image are shown in panel **d**. **(g)** AFM images recorded at different regions within the CSF sample from patient KSSG:19 showed short fibrillar aggregates present together with large spherical particles. Based on AFM sectional analysis of single fibrils detected in CSF from patient KSSG:19 we calculated a mean fibril length of (0.3 ± 0.2) µm and a mean fibril height of (2.7 ± 1.2) nm.

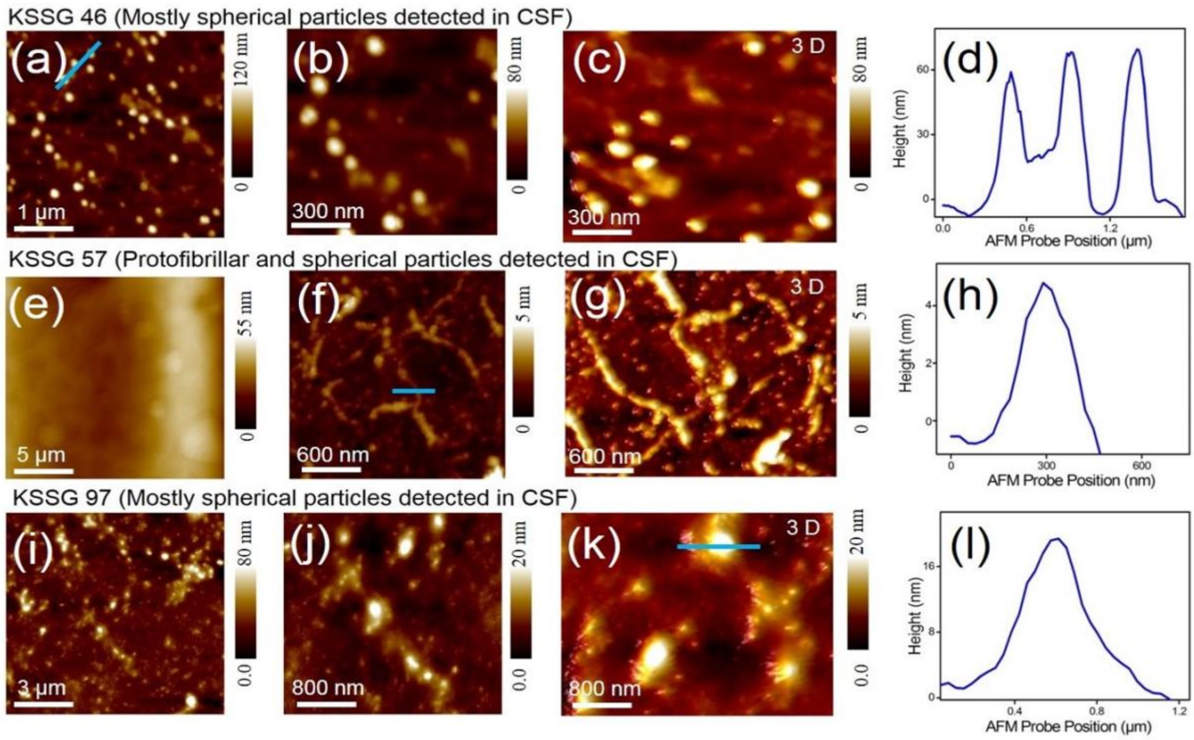

Fig. S10: Morphological analysis of CSF from individuals with SCD (no AD pathology). (a-c) AFM topography shows mostly spherical aggregates in CSF from patient KSSG:46. The size of spherical particles detected in CSF from patient KSSG:46 varied from ~5 to 80 nm. **(d)** Sectional profile extracted along the blue line indicated in panel a across individual spherical particles. No fibrillar aggregates were detected in CSF from patient KSSG:46 during the AFM measurements. **(e-g)** AFM images were recorded in CSF from patient KSSG:57. The AFM images shown in panels **f** and **g** were recorded within areas of the large-area AFM scan (panel **e**). The presence of both spherical particles and protofibrillar segments are visible in the AFM images **(f and g)** recorded on CSF retained in a hydrated state. **(h)** A cross-sectional height profile extracted along the blue line indicated in panel **f** shows the height profile of a single protofibrillar aggregate. The size of the spherical particles detected in CSF from patient KSSG:57 ranged from ~2 to 40 nm. **(i-k**) AFM images were recorded in CSF from patient KSSG:97. The AFM topography revealed mainly the prevalent occurrence of spherical particles with sizes ranging from ~2-30 nm. No fibrillar (protofibrils or mature fibrils) were detected in CSF from patient KSSG:46 and KSSG: 97. **(i)** Cross-sectional height profile extracted along the blue line indicated in panel **k** recorded along with a single spherical particle.

Supplementary References

1 Usov, I. & Mezzenga, R. FiberApp: An Open-Source Software for Tracking and Analyzing Polymers, Filaments, Biomacromolecules, and Fibrous Objects. *Macromolecules* **48**, 1269-1280, (2015).

2 Wegmann, S., Medalsy, I. D., Mandelkow, E. & Müller, D. J. The fuzzy coat of pathological human Tau fibrils is a two-layered polyelectrolyte brush. *Proceedings of the National Academy of Sciences* **110**, E313, (2013).

3 Stark, M., Möller, C., Müller, D. J. & Guckenberger, R. From Images to Interactions: High-Resolution Phase Imaging in Tapping-Mode Atomic Force Microscopy. *Biophys J* **80**, 3009-3018, (2001).
