# Supplementary material for: Protein fibril length in cerebrospinal fluid is increased in Alzheimer’s disease
Source: Commun Biol. 2023 Mar 8;6:251. doi: 10.1038/s42003-023-04606-7 (PMC9995532; doi:10.1038/s42003-023-04606-7)
Supplement: Supplementary file 3 — Description of Additional Supplementary Files [file 42003_2023_4606_MOESM3_ESM.pdf]

Description of Additional Supplementary Files

File name: Supplementary Data 1
Description: The source data for the statistical plots shown in Figures 3, 4 and 5.
