# Supplementary material for: Protein fibril length in cerebrospinal fluid is increased in Alzheimer’s disease
Source: Commun Biol. 2023 Mar 8;6:251. doi: 10.1038/s42003-023-04606-7 (PMC9995532; doi:10.1038/s42003-023-04606-7)
Supplement: Supplementary file 5 — Reporting Summary [file 42003_2023_4606_MOESM5_ESM.pdf]

nature portfolio

Corresponding author(s): Peter Nirmalraj and Ansgar Felbecker

Last updated by author(s): 31/1/2023

Reporting Summary

Nature Portfolio wishes to improve the reproducibility of the work that we publish. This form provides structure for consistency and transparency in reporting. For further information on Nature Portfolio policies, see our Editorial Policies and the Editorial Policy Checklist.

Statistics

For all statistical analyses, confirm that the following items are present in the figure legend, table legend, main text, or Methods section.

n/a	Confirmed	
☐	☒	The exact sample size (*n*) for each experimental group/condition, given as a discrete number and unit of measurement
☐	☒	A statement on whether measurements were taken from distinct samples or whether the same sample was measured repeatedly
☐	☒	The statistical test(s) used AND whether they are one- or two-sided<br>*Only common tests should be described solely by name; describe more complex techniques in the Methods section.*
☐	☒	A description of all covariates tested
☐	☒	A description of any assumptions or corrections, such as tests of normality and adjustment for multiple comparisons
☐	☒	A full description of the statistical parameters including central tendency (e.g. means) or other basic estimates (e.g. regression coefficient) AND variation (e.g. standard deviation) or associated estimates of uncertainty (e.g. confidence intervals)
☐	☒	For null hypothesis testing, the test statistic (e.g. *F*, *t*, *r*) with confidence intervals, effect sizes, degrees of freedom and *P* value noted<br>*Give P values as exact values whenever suitable.*
☒	☐	For Bayesian analysis, information on the choice of priors and Markov chain Monte Carlo settings
☐	☒	For hierarchical and complex designs, identification of the appropriate level for tests and full reporting of outcomes
☐	☒	Estimates of effect sizes (e.g. Cohen's *d*, Pearson's *r*), indicating how they were calculated

Our web collection on statistics for biologists contains articles on many of the points above.

Software and code

Policy information about availability of computer code

Data collection	AFM images were recorded using multimode 8 AFM in tapping mode.
Data analysis	AFM data was analysed using Nanosope software provided by the Bruker companny. The length and junction analysis of the fibril length was done using Fiberapp software. Statistical analyses were performed in the R environment for statistical computing (version 4.2.0).

For manuscripts utilizing custom algorithms or software that are central to the research but not yet described in published literature, software must be made available to editors and reviewers. We strongly encourage code deposition in a community repository (e.g. GitHub). See the Nature Portfolio guidelines for submitting code & software for further information.

Data

Policy information about availability of data

All manuscripts must include a data availability statement. This statement should provide the following information, where applicable:

- Accession codes, unique identifiers, or web links for publicly available datasets
- A description of any restrictions on data availability
- For clinical datasets or third party data, please ensure that the statement adheres to our policy

The datasets generated during and/or analysed during the current study are available from the corresponding author on reasonable request.

Field-specific reporting

Please select the one below that is the best fit for your research. If you are not sure, read the appropriate sections before making your selection.

☒ Life sciences ☐ Behavioural & social sciences ☐ Ecological, evolutionary & environmental sciences

For a reference copy of the document with all sections, see nature.com/documents/nr-reporting-summary-flat.pdf

Life sciences study design

All studies must disclose on these points even when the disclosure is negative.

Sample size	The number of patients included in this study was 33.
Data exclusions	none
Replication	CSF samples were prepared on 2 gold substrates for each patient. And the samples were thoroughly analysed at mutiple sites on the samples using AFM tips cleaned according to protocols described in the paper.
Randomization	*Describe how samples/organisms/participants were allocated into experimental groups. If allocation was not random, describe how covariates were controlled OR if this is not relevant to your study, explain why.*
Blinding	Out of 33 patients included in the study, the analysts were blinded to clinical history of 24 patients. The clinical record of 9 patients was known from a previous study.

Reporting for specific materials, systems and methods

We require information from authors about some types of materials, experimental systems and methods used in many studies. Here, indicate whether each material, system or method listed is relevant to your study. If you are not sure if a list item applies to your research, read the appropriate section before selecting a response.

Materials & experimental systems

n/a	Involved in the study	
☒	☐	Antibodies
☒	☐	Eukaryotic cell lines
☒	☐	Palaeontology and archaeology
☒	☐	Animals and other organisms
☐	☒	Human research participants
☐	☒	Clinical data
☒	☐	Dual use research of concern

Methods

n/a	Involved in the study	
☒	☐	ChIP-seq
☒	☐	Flow cytometry
☐	☒	MRI-based neuroimaging

Human research participants

Policy information about studies involving human research participants

Population characteristics	Overall, seven patients presented with solely subjective cognitive decline (SCD) and one patient had mild cognitive impairment of unspecified etiology. Ten were diagnosed with mild cognitive impairment due to AD (MCI AD) and eleven patients with probable AD dementia with biomarker evidence (ADD) according to the NIA-AA criteria of 2011. One patient was diagnosed with Parkinson's disease dementia (PDD), two with Lewy Body dementia (LBD) and one patient with dementia due to vascular disease (VDD) according to the respective diagnostic criteria.<br>A detailled summary of patient characteristics is given in Table S1. Individual clinical data are presented in Table 1.
Recruitment	In total, 238 patients who presented for an initial or follow-up assessment of cognitive complaints at the memory clinic of the Department of Neurology at the Cantonal Hospital St. Gallen were enrolled in a pilot study on AFM imaging of blood and CSF in neurodegenerative diseases.<br>For the presented analyses, we initially selected nine patients with either AD dementia (n = 3), mild cognitive impairment due to AD (n=1) and patients with only subjective cognitive decline without amyloid pathology (SCD, n = 5) from the well-characterized cohort described in our previous publication 36 to establish the method of AMF analysis of CSF. The nine patients were selected as they span the AD continuum from patients with only subjective cognitive complaints without biomarker evidence for amyloid-, tau-pathology or neurodegeneration (A-T-N-) to severe dementia due to AD (A+T+N+) according to the NIA-AA research framework 42. In a second step, an additional 24 patients were randomly selected from the overall cohort by P.N.N with the clinical information unbeknownst to them, resulting in an analysed cohort of 33 patients.
Ethics oversight	The experimental procedures were approved by the ethics committee of East Switzerland (Number: 202000558) .

Note that full information on the approval of the study protocol must also be provided in the manuscript.

Clinical data

Policy information about clinical studies
All manuscripts should comply with the ICMJE guidelines for publication of clinical research and a completed CONSORT checklist must be included with all submissions.

Clinical trial registration	N/A. The study is not a clinical trial.
Study protocol	N/A. The study is not a clinical trial.
Data collection	N/A. The study is not a clinical trial.
Outcomes	N/A. The study is not a clinical trial.

Magnetic resonance imaging

Experimental design

Design type	Representative MRI scans were included for illustrative purposes only. The manuscript contains no MRI analysis.
Design specifications	*Specify the number of blocks, trials or experimental units per session and/or subject, and specify the length of each trial or block (if trials are blocked) and interval between trials.*
Behavioral performance measures	*State number and/or type of variables recorded (e.g. correct button press, response time) and what statistics were used to establish that the subjects were performing the task as expected (e.g. mean, range, and/or standard deviation across subjects).*

Acquisition

Imaging type(s)	Structural MRI (clinical use)
Field strength	3.0 T, except KSSG 97 (1.5 T)
Sequence & imaging parameters	KSSG 97: Siemens Avanto fit, 1.5 T, 192x192, Repetition time 2200 ms, EchoTime 2.34 ms<br>KSSG 15, 19,38, 46, 57 : Siemens Skyra 3T, 448 x 448, Repetition time 2000 ms, EchoTime 2.34 ms<br>KSSG 36, 2 : Siemens Skyra fit 3T, 256 x 256, Repetition time 2000 ms, EchoTime 2.32 ms
Area of acquisition	whole brain scan

Diffusion MRI ☐ Used ☒ Not used

Preprocessing

Preprocessing software	*Provide detail on software version and revision number and on specific parameters (model/functions, brain extraction, segmentation, smoothing kernel size, etc.).*
Normalization	*If data were normalized/standardized, describe the approach(es): specify linear or non-linear and define image types used for transformation OR indicate that data were not normalized and explain rationale for lack of normalization.*
Normalization template	*Describe the template used for normalization/transformation, specifying subject space or group standardized space (e.g. original Talairach, MNI305, ICBM152) OR indicate that the data were not normalized.*
Noise and artifact removal	*Describe your procedure(s) for artifact and structured noise removal, specifying motion parameters, tissue signals and physiological signals (heart rate, respiration).*
Volume censoring	*Define your software and/or method and criteria for volume censoring, and state the extent of such censoring.*

Statistical modeling & inference

Model type and settings	*Specify type (mass univariate, multivariate, RSA, predictive, etc.) and describe essential details of the model at the first and second levels (e.g. fixed, random or mixed effects; drift or auto-correlation).*
Effect(s) tested	*Define precise effect in terms of the task or stimulus conditions instead of psychological concepts and indicate whether ANOVA or factorial designs were used.*

Specify type of analysis: ☐ Whole brain ☐ ROI-based ☐ Both

Statistic type for inference (See Eklund et al. 2016)	*Specify voxel-wise or cluster-wise and report all relevant parameters for cluster-wise methods.*

Correction

Describe the type of correction and how it is obtained for multiple comparisons (e.g. FWE, FDR, permutation or Monte Carlo).

Models & analysis

n/a	Involved in the study
☒	☐ Functional and/or effective connectivity
☒	☐ Graph analysis
☒	☐ Multivariate modeling or predictive analysis
