# Supplementary material for: Protein fibril length in cerebrospinal fluid is increased in Alzheimer’s disease
Source: Commun Biol. 2023 Mar 8;6:251. doi: 10.1038/s42003-023-04606-7 (PMC9995532; doi:10.1038/s42003-023-04606-7)
Supplement: Supplementary file 1 — Peer Review File [file 42003_2023_4606_MOESM1_ESM.pdf]

nature portfolio

Peer Review File

Open Access This file is licensed under a Creative Commons Attribution 4.0 International License, which permits use, sharing, adaptation, distribution and reproduction in any medium or format, as long as you give appropriate credit to the original author(s) and the source, provide a link to the Creative Commons license, and indicate if changes were made. In the cases where the authors are anonymous, such as is the case for the reports of anonymous peer reviewers, author attribution should be to 'Anonymous Referee' followed by a clear attribution to the source work. The images or other third party material in this file are included in the article's Creative Commons license, unless indicated otherwise in a credit line to the material. If material is not included in the article's Creative Commons license and your intended use is not permitted by statutory regulation or exceeds the permitted use, you will need to obtain permission directly from the copyright holder. To view a copy of this license, visit http://creativecommons.org/licenses/by/4.0/.

This manuscript has been previously reviewed at another Nature Portfolio journal. This document only contains reviewer comments and rebuttal letters for versions considered at Communications Biology.

REVIEWERS' COMMENTS:

Reviewer #1 (Remarks to the Author):

Several of my original questions remain unanswered. The authors have now added several new cases which is an important advancement(!), but the paper still lacks any cases with preclinical AD (i.e. 5 controls or SCD who are A+ or even better A+T+), which is a major limitation considering that most current AD research is focusing on this early AD population (Q1). Further, there is no analyses of brain tissue (Q2). Finally, there is still only indirect evidence that the fibrils consist of Abeta (Q5).
